# Pan-Cancer Analysis Shows That ALKBH5 Is a Potential Prognostic and Immunotherapeutic Biomarker for Multiple Cancer Types Including Gliomas

**DOI:** 10.3389/fimmu.2022.849592

**Published:** 2022-04-04

**Authors:** Cheng Wei, Bo Wang, Dazhao Peng, Xiaoyang Zhang, Zesheng Li, Lin Luo, Yingjie He, Hao Liang, Xuezhi Du, Shenghui Li, Shu Zhang, Zhenyu Zhang, Lei Han, Jianning Zhang

**Affiliations:** ^1^ Tianjin Neurological Institute, Key Laboratory of Post-Neuroinjury Neuro-repair and Regeneration in Central Nervous System, Ministry of Education and Tianjin City, Tianjin Medical University General Hospital, Tianjin, China; ^2^ Department of Neurosurgery, The First Affiliated Hospital of Zhengzhou University, Zhengzhou, China; ^3^ Department of Hepatopancreatobiliary Surgery, The Second Hospital of Tianjin Medical University, Tianjin, China

**Keywords:** ALKBH5, pan-cancer, prognosis, immune, glioma, lncRNA-miRNA-ALKBH5 network

## Abstract

**Background:**

AlkB homolog 5 (ALKBH5) is a N^6^-methyladenosine (m^6^A) demethylase associated with the development, growth, and progression of multiple cancer types. However, the biological role of ALKBH5 has not been investigated in pan-cancer datasets. Therefore, in this study, comprehensive bioinformatics analysis of pan-cancer datasets was performed to determine the mechanisms through which ALKBH5 regulates tumorigenesis.

**Methods:**

Online websites and databases such as NCBI, UCSC, CCLE, HPA, TIMER2, GEPIA2, cBioPortal, UALCAN, STRING, SangerBox, ImmuCellAl, xCell, and GenePattern were used to extract data of ALKBH5 in multiple cancers. The pan-cancer patient datasets were analyzed to determine the relationship between ALKBH5 expression, genetic alterations, methylation status, and tumor immunity. Targetscan, miRWalk, miRDB, miRabel, LncBase databases and Cytoscape tool were used to identify microRNAs (miRNAs) and long non-coding RNAs (lncRNAs) that regulate expression of ALKBH5 and construct the lncRNA-miRNA-ALKBH5 network. *In vitro* CCK-8, wound healing, Transwell and M2 macrophage infiltration assays as well as *in vivo* xenograft animal experiments were performed to determine the biological functions of ALKBH5 in glioma cells.

**Results:**

The pan-cancer analysis showed that ALKBH5 was upregulated in several solid tumors. ALKBH5 expression significantly correlated with the prognosis of cancer patients. Genetic alterations including duplications and deep mutations of the *ALKBH5* gene were identified in several cancer types. Alterations in the *ALKBH5* gene correlated with tumor prognosis. GO and KEGG enrichment analyses showed that ALKBH5-related genes were enriched in the inflammatory, metabolic, and immune signaling pathways in glioma. ALKBH5 expression correlated with the expression of immune checkpoint (ICP) genes, and influenced sensitivity to immunotherapy. We constructed a lncRNA-miRNA network that regulates ALKBH5 expression in tumor development and progression. *In vitro* and *in vivo* experiments showed that ALKBH5 promoted proliferation, migration, and invasion of glioma cells and recruited the M2 macrophage to glioma cells.

**Conclusions:**

ALKBH5 was overexpressed in multiple cancer types and promoted the development and progression of cancers through several mechanisms including regulation of the tumor-infiltration of immune cells. Our study shows that ALKBH5 is a promising prognostic and immunotherapeutic biomarker in some malignant tumors.

## Introduction

Nucleoside modifications affect the biological functions and stability of the RNA molecules. According to MODOMICS, the database of RNA modifications, 163 different nucleoside modifications have been reported for RNA molecules in all organisms (1). The most abundant RNA modification is N^6^-methyladenosine (m^6^A), which involves methylation of the 6^th^ nitrogen atom of the adenine (A) molecule in either messenger RNAs (mRNAs) or non-coding RNAs (ncRNAs) (2, 3). The m^6^A modification is catalyzed by the m^6^A methylation writer complex consisting of several proteins including METTL3 and METTL14, and is reversed by demethylase erasers such as FTO and ALKBH5 (4). The m^6^A modification is recognized by m^6^A reader proteins belonging to the YTHDF and YTHDC protein families, and modulates translation, localization, mRNA splicing, or stability of the modified mRNA or ncRNA (5). Several studies have shown that aberrant regulation of m^6^A RNA modifications is associated with the development and progression of various cancers (6–8).

AlkB homolog 5 (ALKBH5) is a widely reported m^6^A RNA demethylase that belongs to the AlkB family of DNA and RNA demethylases, which mediates the repair of N-alkylated nucleobases by oxidative demethylation and consists of 9 mammalian homologs (9). The full-length ALKBH5 has 394 amino acids and ALKBH5 mainly locates on nuclear speckles that facilitate the assembly of mRNA-processing factors, which sustains the view that nuclear nascent RNAs are the main substrates of ALKBH5. ALKBH5 plays a significant role in several biological processes such as cell proliferation, osteogenesis, spermatogenesis, and ossification (10–13). ALKBH5 also plays an oncogenic or tumor suppressive role in different kinds of cancers, such as breast cancer, glioblastoma, ovarian cancer, lung cancer, liver cancer, pancreatic cancer, gastrointestinal cancer, acute myeloid leukemia (AML) and osteosarcoma (14–20). On the one hand, breast cancer stem cell (BCSC) phenotype could be induced by hypoxia through HIF-dependent and ALKBH5-mediated regulation of NANOG mRNA (14). And ALKBH5 was highly expressed in glioblastoma stem-like cells (GSCs) and maintained tumorigenic activity of GSCs *via* maintaining FOXM1 expression and cell proliferation program (15). Hu et al. found that ALKBH5 promoted epithelial ovarian cancer cell proliferation and invasion by inhibiting autophagy through miR-7 and Bcl-2 (16). Chen et al. discovered that ALKBH5 was essential for the development and maintenance of AML and the self-renewal of leukemia stem/initiating cells (LSCs/LICs), and ALKBH5 played a tumorigenic role in AML through post-transcriptional regulation of its key targets, such as TACC3 (21). On the other hand, ALKBH5 inhibited tumor growth and metastasis *via* decreasing YTHDFs-mediated YAP expression and suppressing miR-107/LATS2-mediated YAP activity (17). Zheng et al. found that ALKBH5-mediated m6A demethylation led to post-transcriptional inhibition of LY6/PLAUR Domain Containing 1 (LYPD1), which inhibited malignant progression of hepatocellular carcinoma (22). In osteosarcoma, ALKBH5 could inhibit tumor progression through epigenetic silencing of m6A-dependent pre-miR-181B-1/YAP signaling pathway (20). Furthermore, Tariq M Rana revealed that ALKBH5 regulated the response of anti-PD-1 therapy by regulating lactate in tumor microenvironment and inhibiting immune cell recruitment (23). These results indicate that altered ALKBH5 expression could both promote and suppress carcinogenesis based on cancer type and ALKBH5 acts as a significant role in tumor immune microenvironment. At present, there are few systematic studies on ALKBH5 in pan-cancer, especially in glioma.

In this study, we performed systematic bioinformatics analysis to determine the biological functions and prognostic significance of ALKBH5 in several cancers using the patient data in multiple databases. The functional significance of altered expression levels of ALKBH5 was comprehensively investigated in various cancers in regard to prognosis, genetic alterations, expression levels of ALKBH5-related genes, tumor immunity, and the lncRNA-miRNA network regulating ALKBH5 expression. We also specifically analyzed the relationship between ALKBH5 expression and the growth and progression of gliomas using both *in vitro* and *in vivo* models.

## Materials and Methods

### Analysis of ALKBH5 Gene and Protein Expression

The Human Protein Atlas (HPA) online website (https://www.proteinatlas.org/) was used to analyze ALKBH5 in “Tissue Atlas”. The expression data of ALKBH5 gene in different human normal tissues were obtained (24). The row data source was TMM normalized. Normalized eXpression (NX), the resulting transcript expression values, were calculated for each gene in every sample. Online website (https://www.proteinatlas.org/about/assays+annotation) showed the detailed information. NX ≥ 1 was regarded as “Low specificity” in at least one tissue, but not increased in other tissue. Then, the Cancer Genome Atlas (TCGA) cohort was used to analyze ALKBH5 expression in 33 tumors, including adrenocortical carcinoma (ACC), bladder urothelial carcinoma (BLCA), breast invasive carcinoma (BRCA), cervical squamous cell carcinoma and endocervical adenocarcinoma (CESC), cholangiocarcinoma (CHOL), colon adenocarcinoma (COAD), lymphoid neoplasm diffuse large B-cell lymphoma (DLBC), esophageal carcinoma (ESCA), glioblastoma multiforme (GBM), head and neck cancer (HNSC), kidney chromophobe (KICH), kidney renal clear cell carcinoma (KIRC), kidney renal papillary cell carcinoma (KIRP), acute myeloid leukemia (LAML), brain lower grade glioma (LGG), liver hepatocellular carcinoma (LIHC), lung adenocarcinoma (LUAD), lung squamous cell carcinoma (LUSC), Mesothelioma (MESO), ovarian serous cystadenocarcinoma (OV), pancreatic adenocarcinoma (PAAD), pheochromocytoma and paraganglioma (PCPG), prostate adenocarcinoma (PRAD), rectum adenocarcinoma (READ), sarcoma (SARC), skin cutaneous melanoma (SKCM), stomach adenocarcinoma (STAD), testicular germ cell tumors (TGCT), thyroid carcinoma (THCA), thymoma (THYM), uterine corpus endometrial carcinoma (UCEC), uterine carcinosarcoma (UCS) and uveal melanoma (UVM) *via* Gene Expression Profiling Interactive Analysis 2.0 (GEPIA2) (http://gepia2.cancer-pku.cn/#index) (25). All abbreviations showed in **Supplementary Table 1**. Using TCGA combined with Genotype Tissue Expression (GTEx) cohort, ALKBH5 expression was analyzed between tumors and corresponding normal tissues in 27 tumors *via* SangerBox (http://SangerBox.com/Tool) (26). ALKBH5 mRNA expression data of glioma with different gardes, subtypes and new types (oligodendroglioma, astrocytoma and glioblastoma) were obtained from the Chinese Glioma Genome Atlas (CGGA) (http://www.cgga.org.cn; dataset ID: mRNAseq_325 and mRNAseq_693) and TCGA databases.

Moreover, RNA-Seq data (lllumina HiSeq X Ten, Novogene) and corresponding pathological and clinical data of external 100 glioma samples were obtained to further validate the correlation between ALKBH5 mRNA expression and glioma grades or prognosis. All human glioma samples were taken from patients undergoing surgery at the First Affiliated Hospital of Zhengzhou University. Tissue samples were graded by neuropathologists according to World Health Organization (WHO) standards and stored in liquid nitrogen. Glioma specimens were divided into grade II (30 cases), grade III (20 cases) and grade IV (50 cases). Histological and clinical data of glioma specimens are shown in **Supplementary Table 2**. This study was approved by the institutional review boards of the hospital and obtained written informed consent from all patients.

In addition, the ALKBH5 protein expression data for 44 tissues under physiological conditions were obtained in HPA portal. The subcellular localization information of ALKBH5 was obtained using the Cell Atlas module. The conserved functional domains of ALKBH5 proteins were analyzed in different species using “HomoloGene” function of NCBI database. The expression levels of ALKBH5 protein were also analyzed in Clinical Proteomic Tumor Analysis Consortium (CPTAC) module *via* UALCAN portal (http://ualcan.path.uab.edu/index.html) (27). The CPTAC database normalized the expression values in each proteome data to the standard deviation of the median. The total ALKBH5 protein expression level was compared between tumors and normal tissues *via* retrieving “ALKBH5”. Five available data sets for tumors were selected, including OV, breast cancer, clear cell RCC (ccRCC), lung adenocarcinoma and UCEC.

### Survival Prognosis Analysis

The overall survival (OS) and disease-free survival (DFS) survival map data for ALKBH5 in various tumor types in TCGA database were obtained *via* GEPIA2 online website. The high-expression and low-expression cohorts of ALKBH5 was obtained through the expression threshold of the cutoff-high (50%) and cutoff-low (50%) values. The “Survival Analysis” module of GEPIA2 was used to analyze special survival plots with log-rank P-values.

Then, the COX_OS (overall survival) and COX_DSS (disease specific survival) analysis data of ALKBH5 for different tumors was analyzed on SangerBox portal. In Kaplan-Meier Plotter website (https://kmplot.com/analysis/), the relationship between ALKBH5 expression and prognosis of tumor patients, such as OS, progression free survival (PFS), first progression (FP), post progression survival (PPS) and relapse free survival (RFS), was analyzed (28). The Kaplan-Meier survival plots of ovarian, lung, gastric and liver cancer cases were generated in the “mRNA gene chip” and “mRNA RNA-seq” modules. The log-rank P-value, hazard ratio (HR) and 95% confidence intervals were computed. Through CGGA mRNAseq_325 (CGGA_325), CGGA mRNAseq_693 (CGGA_693) and TCGA mRNAseq databases, the relationship between ALKBH5 and overall survival of glioma patients in primary glioma, recurrent glioma, LGG (WHO I-III) and GBM (WHO IV) was analyzed. Through these three databases, the relationship between ALKBH5 expression and prognosis in IDH mut+1p/19q codeletion, IDH mut+1p/19q noncodeletion and IDH wild groups was analyzed in glioma. The relationship between ALKBH5 expression and prognosis in IDH status, 1p/19q codeletion status and MGMT promoter status was also analyzed in glioma *via* CGGA_325 and CGGA _693 datasets. Cox regression analysis of CGGA_325 combined with CGGA _693 and TCGA datasets in glioma was performed *via* SPSS and nomogram was used to visualize the results *via* SangerBox.

### Genetic Alteration Analysis

Firstly, the genetic mutation ratios of *ALKBH5* in various tumors was explored *via* the “Gene_Mutation” module of the Tumor Immune Estimation Resource 2.0 (TIMER2) website (http://timer.cistrome.org/) (29). The row data represented the percentages of *ALKBH5* mutation in tumors.

Then, the characteristics of *ALKBH5* genetic alterations were explored by the online cBioPortal database (https://www.cbioportal.org/) (30). TCGA Pan Cancer Atlas Studies in the “Quick select” section was selected for query. The mutation type, alteration frequency, and copy number alteration (CNA) data were observed across TCGA tumor datasets in the “Cancer Types Summary” module.

Databases of somatic mutations and somatic copy number alternations (CNAs) were obtained from TCGA datasets. CNAs correlated with ALKBH5 expression, and the threshold copy number at alteration peaks were analyzed by GISTIC 2.0 (https://cloud.genepattern.org/) (31). The patients were divided into the first 25% ALKBH5^high^ (n=166) and the last 25% ALKBH5^low^ (n=166) groups according to the expression value of ALKBH5. The maftools package was also used in R software (https://www.r-project.org/) to download and visualize the somatic mutations of patients with 25% ALKBH5^high^ and 25% ALKBH5^low^ glioma across TCGA databases (32).

### ALKBH5-Related Gene Enrichment Analysis

Through searching the STRING website (https://string-db.org/), “ALKBH5” was queried in the “protein name” module and “*Homo sapiens*” in the organism module (33). The following main parameters was set: meaning of network edges (“evidence”), minimum required interaction score [“low confidence (0.150)”], active interaction sources (“experiments” and “database”) and maximum number of interactors to show (“no more than 50 interactors” in the 1st shell). The available ALKBH5-binding proteins was analyzed. Then, the top 10 ALKBH5-associated targeting genes in TCGA tumors were generated by the “Similar Gene Detection” module of GEPIA2. The “correlation analysis” module of GEPIA2 was used to explore the correlation between ALKBH5 and these 10 ALKBH5-associated targeting genes. The P-value and the correlation coefficient (R) were generated. The correlation scatter plot of 10 genes was generated *via* the log_2_ TPM. We obtained a heatmap of these top 9 targeting genes *via* the “Gene_Corr” function of TIMER2.

Analyses of ALKBH5 related genes in CGGA_325, CGGA_693 and TCGA databases were performed using R package. The first 100 genes positively or negatively correlated with ALKBH5 expression were screened in CGGA_325, CGGA_693 and TCGA databases, and a total of 200 genes were used for subsequent analysis. SangerBox portal was used to analyze and map the results of GO-BP analysis of ALKBH5 related genes in glioma. Then, GSE93054 database (https://www.ncbi.nlm.nih.gov/geo/query/acc.cgi?acc=GSE93054) was used to analyze the biological functions of ALKBH5 related genes in glioma stem cells (GSCs). In GSC11 and GSC17 cells, 3032 and 5158 differentially expressed genes were found after knockdown ALKBH5, respectively. Among them, 1150 differentially expressed genes were intersected within GSC11 and GSC17 cell lines. GO-BP and KEGG enrichment analyses of ALKBH5 related genes was analyzed and mapped in glioma by SangerBox portal.

### Analysis of Immune Cell Infiltration

The relationships between ALKBH5 expression and ESTIMATE score and tumor infiltration immune cells (TIICs) in multiple tumors were explored *via* the SangerBox website, including CD4+ T cells, CD8+ T cells, B cells, neutrophils, macrophages, etc. XCell (https://xcell.ucsf.edu/) and ImmuCellAI (http://bioinfo.life.hust.edu.cn/ImmuCellAI#!/) portals were used to analyze the relationship between ALKBH5 expression and immune-related cells in glioma *via* CGGA_325, CGGA_693 and TCGA datasets (34, 35).

Metagene analysis was performed with gene set variation analysis (GSVA) using the R package GSVA. Metagenes included HCK, IgG, Interferon, LCK, MHC-I, MHC-II and STAT1 clusters (**Supplementary Table 3**). The relationships between ALKBH5 expression and microsatellite instability (MSI), tumor mutation burden (TMB), Neoantigens and immune checkpoint (ICP) in different tumors from TCGA cohorts were investigated *via* the SangerBox website. These checkpoint genes included ADORA2A, BTLA, BTNL2, CD160, CD200, CTLA4, HHLA2, ICOS, IDO1, LAG3, PDCD1, TNFRSF14, VTCN1, etc. Spearman’s rank correlation test was performed, and the partial correlation (cor) and P-value were generated. Details of immune checkpoint receptor and ligand genes shown in **Supplementary Table 4**.

### LncRNA-miRNA-ALKBH5 Regulatory Network Analysis

Upstream binding miRNAs of ALKBH5 were predicted by several target gene prediction programs, consisting of miRWalk (http://mirwalk.umm.uni-heidelberg.de/), miRDB (http://mirdb.org/), miRabel and TargetScan (http://www.targetscan.org/vert_72/) (36–39). Only the 49 predicted miRNAs that appeared in these four programs were included for subsequent analyses. Then, top 10 miRNAs ranked in TargetScan program were regarded as candidate miRNAs of ALKBH5, including miR-4732-5p, miR-193a-3p, miR-362-3p, miR-193b-3p, miR-589-5p, miR-4736, miR-6840-3p, miR-329-3p, miR-5008-5p and miR-6132. The CGGA microRNA_array_198 cohort was used to analyze the clinical features of the top 10 miRNAs. In addition, upstream target lncRNAs by miRNA screened were predicted and analyzed in “Experimental module” and “Prediction module” *via* LncBase database (http://carolina.imis.athena-innovation.gr/diana_tools/web/index.php?r=lncba sev2%2Findex) (40). Subsequently, Cytoscape software was applied to visualize the lncRNA-miRNA-ALKBH5 regulatory network.

### Cell Lines and Culture

Human astrocytes (HA) and human glioma cell lines (U87, LN229, U251, A172 and B19) were obtained from ATCC (American Type Culture Collection, Manassas, VA, USA). Human THP-1 cells were purchased from the Shanghai Cell Bank of the Chinese Academy of Sciences (Shanghai, China). All cell line authentication service was applied and mycoplasma was also routinely tested. Astrocyte medium (AM) (Cat. #1801, ScienCell, USA) was used to culture human astrocytes, supplemented with 10 mL of fetal bovine serum (FBS) (Cat. #0010, ScienCell, USA), 5 mL of penicillin/streptomycin solution (P/S) (Cat. #0503, ScienCell, USA) and 5 mL of Astrocyte Growth Supplement (AGS) (Cat. #1852, ScienCell, USA). The glioma cell lines were cultured in DMEM Medium (Dulbecco’s Modified Eagle Medium, Gibco, USA) and THP-1 cells were cultured in RPMI 1640 medium with 10% FBS (FBS, BI serum, Israel) and cultured in a 37°C constant temperature incubator containing 5% CO_2_.

### SiRNAs or Plasmids Delivery, Total RNA Isolation, Reverse Transcription and Quantitative Real-Time Polymerase Chain Reaction

The ALKBH5 siRNAs were purchased from Shanghai GenePharma Co., Ltd. SiRNAs were applied to knockdown the expression of ALKBH5 in U87 and U251 cell lines and transfected *via* Lipofectamine ™ RNAiMax (13778150, Thermo Fisher Scientific, USA) reagent. The ALKBH5 overexpression plasmids (pEGFP-C1B-ALKBH5) were kindly supplied by professor Xudong Wu (Tianjin Medical University, China) and transfected into cell lines *via* Lipofectamine 3000. RT-qPCR was applied to detect the efficiency of ALKBH5 knockdown or overexpression. Glioma cells were planted in 6-well plates, 5μL Lipofectamine ™ RNAiMAX and 40 pmol siRNA were mixed in each well for 5 min. Then, mixture was added into the cells for transfection. After transfection, the cells were replaced with full medium at 24h. The cells were collected after 48h. The total RNA was extracted with TRIzol reagent (Thermo Fisher Scientific, Inc.). The GoScript reverse transcription system (Promega Corporation) was used to synthesize cDNA. The mRNA expression status was detected *via* GoTaq^®^qPCR Master Mix (Promega Corporation) on ABI QuantStudio 3. GAPDH was applied as an internal control. The oligonucleotide primers used for quantitative PCR were as follows: ALKBH5, 5’- GGACCCCATCCACATCTTCG-3’ forward and 5’-GGCCGTATG CAGTGAGTGAT-3’ reverse; GAPDH, 5’-GGTGGTCTCCTCTGACTTCAACA-3’ forward and 5’-GTTGCTGTAGCCAAA TTCGTTGT-3’ reverse. The following PCR program was used: 95˚C for 10 min, 40 cycles of amplification (15 sec at 95˚C, 40 sec at 60˚C and 1 min at 72˚C) and a final extension step (72˚C for 2 min). All PCR experiments were performed in triplicate. The siRNA sequences were shown below: siALKBH5#1, 5’-CUGCGCAACAAGUACUUCUTT-3’sense and 5’-AGAAGUACUUGUUG CGCAGTT-3’ antisense; siALKBH5#2, 5’-CCUCAGGAAGACAA GAUUATT-3’sense and 5’-UAAUCUUGUCUUCCUGAGGTT-3’ antisense; negative control (NC), 5’-UUCUCCGAACGUG UCACGUTT-3’sense and 5’-ACGUGACACGUUCGGAGAATT-3’ antisense.

### CCK-8 Experiment

The CCK-8 reagent was purchased from APExBIO (USA). The 2×10^3^ cells were seeded into 96-well plates in advance. The test was started 24 hours later and lasted for 6 consecutive days (0, 1, 2, 3, 4, 5, 6 days). The medium was absorbed and discarded, and CCK-8 reagent was mixed with serum-free medium in a 5ml eppendorf tube (CCK8 reagent: Serum-free medium =10 μL: 90 μL per well) and added into 96-well plates with 100 μL per well. OD value at 450 wavelength was detected on a microplate after incubation at 37°C for 1h.

### Wound Healing and Transwell Experiments

Wound healing assay: According to the density of 2.5×10^5^ cells per well, U87, U251 and LN229 cells were inoculated in the 6-well plate. Then, siALKBH5#1, siALKBH5#2 or ALKBH5 plasmids were transfected into the 6-well plate. After 24 h, 200 μL pipetting head was used to scratch the cells in the plate and the serum-free medium was replaced to collect images under an inverted microscope (IX81, Olympus Company, Japan) at 0 h, 12h and 24 h.

Transwell assay: The upper chamber of Transwell assay was coated with a layer of Matrigel matrix glue (Corning Company, USA) (matrix glue: Serum-free medium=1:4). Then, the cells were resuspended and counted using serum-free medium. The cells were seeded into the upper chamber at a density of 5×10^4^, and the lower chamber was added 500 μL full medium. After 24h, the cells were fixated with 4% paraformaldehyde for 10 min, 1% crystal violet was used to staining cells for 10 s. The remaining cells in the upper chamber were slightly wiped off, and images were collected under a positive microscope (BX53, Olympus Company, Japan). In addition, the number of cells passing through the chamber was counted in four random fields under the microscope.

### M2 Macrophage Infiltration Assay

THP-1 cells were differentiated into macrophages induced by 150 nM phorbol 12-myristate 13-acetate (PMA; Sigma, USA) with 24 h. Then, macrophage M2 polarization was obtained by incubation with 20 ng/ml of interleukin 4 (R&D Systems, #204-IL) and 20 ng/ml of interleukin 13 (R&D Systems, #213-ILB) for 48 h. M2 macrophage infiltration assays were applied through seeding 1.0×10^5^ M2 macrophage cells (300μl) without serum in the upper chamber of a Transwell plate for 48 h (size 5mm, Corning, NY, USA). In bottom plate, U87 and U251 glioma cells (1.0×10^5^) were cultured with10% FBS in DMEM (700μl). After incubation for 48 h, the cells in the upper chamber were fixed with 4% paraformaldehyde for 10 min and stained with 0.1% crystal violet for 10s. The infiltrated M2 macrophage cells were counted in three randomly selected fields from each membrane.

### Subcutaneous Xenograft Experiment

All mouse experiments were conducted in accordance with protocols approved by the Tianjin medical university animal care and use committee and followed guidelines for animal welfare. Four-week-old BALB/c female nude mice were purchased from Beijing HFK Bioscience Co.,LTD. The tumor masses were subcutaneously embedded in nude mice and the nude mice were randomly divided into two groups (n=5) when the tumor masses grew to 4 to 5mm in diameter. These two groups were control group and siALKBH5 group. After that, tumor volumes were measured every 2 days and ALKBH5 siRNA were injected into the siALKBH5 group at a dose of 10 μL of siRNA versus 10 μL of Lipofectamine ™ 3000 per nude mouse. After 21 days, mice were euthanatized, subcutaneous tumors were removed and images were collected. The tumor volume was calculated with the formula Volume= (length × width^2^)/2.

### Statistical Analysis

Through the online databases mentioned above, the statistical analysis was automatically computed in this study. These results were considered as statistically significant at *P<0.05, **P<0.01, ***P<0.001 and ****P<0.0001.

## Results

### ALKBH5 Expression Is Upregulated in Multiple Tumors Including Gliomas

**Figure 1:**
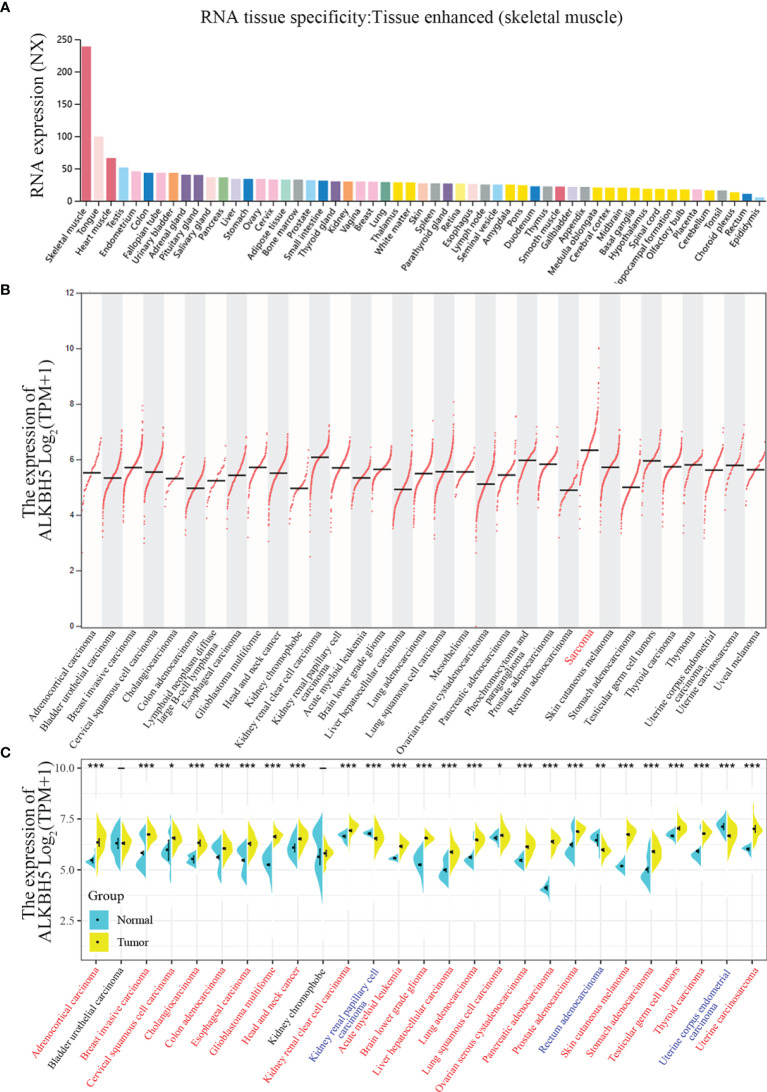
ALKBH5 expression levels in normal tissues and cancers. **(A)** ALKBH5 consensus Normalized eXpression (NX) levels for 54 normal tissue types and 7 blood cell types, generated by the three transcriptomics datasets (GTEx, HPA and FANTOM5); **(B)** ALKBH5 mRNA expression levels in 33 different tumor types from TCGA database *via* GEPIA2 portal (Sarcoma with the highest expression is shown in red label); **(C)** ALKBH5 mRNA expression levels in different tumors and corresponding normal tissues from TCGA and GTEx database by SangerBox. Red label tumors represented up-regulation of ALKBH5 compared with corresponding normal tissues, blue label tumors represented down-regulation of ALKBH5 compared with corresponding normal tissues, and black label tumors represented no significant difference of ALKBH5 between tumor tissues and corresponding normal tissues or absence of normal samples. *P<0.05, **P<0.01, ***P<0.001.

The flowchart of this study is shown in **Supplementary Figure 1**. The normalized expression (NX) levels of ALKBH5 were analyzed in various tumor tissues and their corresponding adjacent normal tissues as well as various tumor cells and the corresponding non-tumor cells in The Human Protein Atlas (THPA) database. ALKBH5 mRNA expression levels were higher in the normal human skeletal muscle, heart muscle, and tongue tissues (NX>50; **Figure 1A**). In most other normal human tissues, ALKBH5 mRNA expression level was detectable (NX>10) but low (NX<50) (**Figure 1A**). Moreover, TCGA database analyses of tumor tissues from 33 cancer types showed that ALKBH5 mRNA expression levels were tumor-specific and had the highest expression in Sarcoma (**Figure 1B**). TCGA and GTEx database analyses showed that the ALKBH5 mRNA levels were upregulated in the adrenocortical carcinoma (ACC), breast invasive carcinoma (BRCA), cervical squamous cell carcinoma and endocervical adenocarcinoma (CESC), cholangiocarcinoma (CHOL), colon adenocarcinoma (COAD), esophageal carcinoma (ESCA), glioblastoma multiforme (GBM), head and neck cancer (HNSC), kidney renal clear cell carcinoma (KIRC), acute myeloid leukemia (LAML), brain lower grade glioma (LGG), liver hepatocellular carcinoma (LIHC), lung adenocarcinoma (LUAD), lung squamous cell carcinoma (LUSC), ovarian serous cystadenocarcinoma (OV), pancreatic adenocarcinoma (PAAD), prostate adenocarcinoma (PRAD), skin cutaneous melanoma (SKCM), stomach adenocarcinoma (STAD), testicular germ cell tumors (TGCT), thyroid carcinoma (THCA) and uterine carcinosarcoma (UCS) tumor tissues compared to the corresponding normal tissues, while decreased in kidney renal papillary cell carcinoma (KIRP), rectum adenocarcinoma (READ) and uterine corpus endometrial carcinoma (UCEC) (All abbreviations showed in **Supplementary Table 1**) (**Figure 1C**).

**Figure 2:**
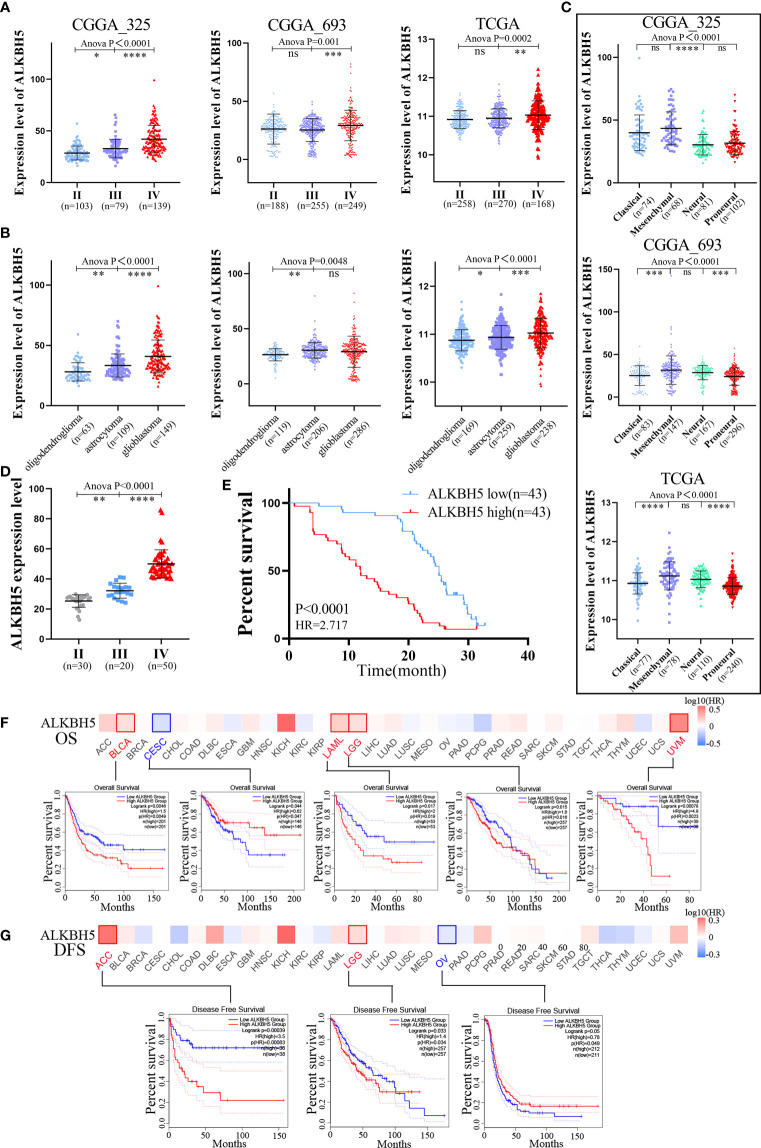
Clinical and molecular characteristics of ALKBH5 in pan-cancer, including gliomas. **(A)** The correlation between ALKBH5 expression and glioma WHO grade (II, II and IV) in CGGA_325, CGGA_693 and TCGA datasets; **(B)** The correlation between ALKBH5 expression and glioma three types (oligodendroglioma, astrocytoma and glioblastoma) in CGGA_325, CGGA_693 and TCGA datasets; **(C)** The relationship between ALKBH5 expression and glioma subtypes (classical, mesenchymal, neural, and proneural) in CGGA_325, CGGA_693 and TCGA datasets; **(D)** The correlation between ALKBH5 expression and grades in 100 glioma samples, including grade II (n=30), grade III(n=20) and grade IV (n=50) samples; **(E)** Overall survival (OS) of different ALKBH5 expression level in 100 glioma samples. The GEPIA2 tool was used to perform OS **(F)** and disease-free survival (DFS) **(G)** analyses of different tumors in TCGA by ALKBH5 expression. Red label indicated positively correlated, blue label indicated negatively correlated. *P<0.05, **P<0.01, ***P<0.001, ****P<0.0001.

Next, we analyzed ALKBH5 mRNA expression levels in different WHO grades, subtypes, and new types of gliomas. Glioblastoma is classified into four subtypes (classical, mesenchymal, neural, and proneural) based on the molecular signatures (41). The gliomas are classified into the following three types according to the fifth edition of the World Health Organization (WHO) classification of tumors of the Central Nervous System (CNS) (WHO CNS5): IDH-mutant and 1p/19q-codeleted (mut+codel) oligodendroglioma, IDH-mutant (mut+non-codel) astrocytoma, and IDH-wildtype (IDH-wild) glioblastoma (42). CGGA and TCGA database analyses showed that ALKBH5 expression levels were positively associated with glioma grades (**Figure 2A**). Moreover, the expression levels of ALKBH5 mRNA correlated with the WHO CNS5 types and glioma subtypes (**Figures 2B, C**). Analysis of 100 glioma cases collected also showed that ALKBH5 expression level was positively correlated with glioma grade, which was consistent with our analysis results in CGGA and TCGA databases (**Figure 2D**). Isocitrate dehydrogenase (IDH) mutations and chromosomal 1p/19q codeletions are associated with better survival outcomes of glioma patients (43). Furthermore, promoter methylation status of the O6-methylguanine DNA methyltransferase (MGMT) is a prognostic indicator of the clinical response to treatment of glioblastoma patients with temozolomide (TMZ) (44). Then, we explored the relationship between ALKBH5 mRNA expression and the status of *IDH* gene mutations, 1p/19q codeletion, and MGMT promoter methylation. Analysis of the TCGA database and the CGGA_325, CGGA_693 datasets showed significantly higher ALKBH5 mRNA levels in the glioma patients with wild-type IDH and chromosomal 1p/19q non-codeletion, and significantly reduced in the glioma patients with MGMT promoter methylation (**Supplementary Figures 2A–C**).

Finally, we then investigated the expression characteristics of the ALKBH5 protein in 43 different normal tissues and various cancers using the THPA database, respectively. ALKBH5 protein expression was higher in most normal tissues and lower in soft tissues (**Supplementary Figure 3A**). ALKBH5 protein was localized in the nucleoplasm, cytosol, and golgi apparatus (**Supplementary Figure 3B**). NCBI database analysis showed that ALKBH5 protein was conserved in multiple species (**Supplementary Figure 3C**). CPTAC database analysis showed that ALKBH5 protein levels were significantly increased in the ovarian cancer, breast cancer, clear cell renal cell carcinoma (ccRCC), and lung adenocarcinoma tissues, and significantly reduced in the uterine corpus endometrial carcinoma (UCEC) tissues compared with the corresponding normal tissues (**Supplementary Figure 3D**).

In summary, our results showed that ALKBH5 mRNA and protein levels were upregulated in several tumors. Furthermore, ALKBH5 expression levels correlated with the grades, subtypes, and clinical features of gliomas.

### ALKBH5 Expression Is Associated With the Prognosis of Various Tumors Including Gliomas

The prognostic value of ALKBH5 expression levels in multiple cancers was analyzed by comparing the rates of overall survival (OS), progression free survival (PFS), disease free survival (DFS), post progression survival (PPS), first progression (FP), and relapse free survival (RFS) in the cancer patients with high or low ALKBH5 expression levels.

GEPIA2 database analysis of the pan-cancer cohorts showed that OS was significantly reduced in BLCA (HR=1.5), LAML (HR=2.0), LGG (HR=1.6), and UVM (HR=4.9) patients with high ALKBH5 mRNA expression levels and significantly increased in CESC (HR=0.62) patients with high ALKBH5 mRNA expression levels (**Figure 2F**). The results showed that patients with high ALKBH5 mRNA expression level had a worse prognosis (OS) than those with low ALKBH5 mRNA expression in BLCA, LAML, LGG and UVM. Furthermore, DFS was significantly reduced in the ACC (HR=3.5) and LGG (1.4) patients with high ALKBH5 mRNA expression levels and significantly increased in the OV (HR=0.78) patients with high ALKBH5 mRNA expression levels (**Figure 2G**). The results suggested that patients with high ALKBH5 mRNA expression level had a worse prognosis (DFS) than those with low ALKBH5 mRNA expression in ACC and LGG. Cox regression analysis of the SangerBox database showed that ALKBH5 mRNA expression levels were associated with OS and disease specific survival (DSS) of patients with multiple cancers. The results showed that the high mRNA expression of ALKBH5 was associated with shorter OS in KICH (HR=7.29), LGG (HR=3.42), LAML (HR=2.11), GBM (HR=1.60) and BLCA (HR=1.40) and shorter DSS in KICH (HR=10.50), LGG (HR=3.23), GBM (HR=1.56) and BLCA (HR=1.42) (**Supplementary Figures 4A, B**). Furthermore, integrated analysis of GEO, EGA, and TCGA databases in the Kaplan-Meier Plotter website showed that ALKBH5 mRNA expression was negatively associated with the OS and PFS rates of ovarian cancer patients, OS, FP and PPS rates of gastric cancer patients, and OS and PPS rates of lung cancer patients (**Supplementary Figures 4C–E**). ALKBH5 mRNA expression showed positive association with OS, PFS, and RFS rates of liver cancer patients (**Supplementary Figure 4F**).

The correlation between ALKBH5 mRNA expression levels and the prognosis of patients with primary glioma, recurrent glioma, low grade glioma (LGG) or glioblastoma (GBM) was investigated using the CGGA and TCGA datasets. In the CGGA_325, CGGA_693, and TCGA datasets, high ALKBH5 expression levels were associated with shorter OS in primary glioma and LGG patients (**Supplementary Figures 5A–C**). Analysis of 100 glioma cases also showed that glioma patients with high ALKBH5 mRNA expression levels had shorter OS compared with patients with low ALKBH5 expression level (**Figure 2E**). Then, Cox regression analyses demonstrated that tumor grade, chemotherapy, IDH mutation status, 1p/19q codeletion, and ALKBH5 mRNA expression levels were independent prognostic predictors based on the CGGA_325 combined with CGGA_693 dataset (**Supplementary Figure 6A**). In TCGA database, tumor grade, age of patients, IDH mutation status and 1p/19q codeletion were independent prognostic predictors (**Supplementary Figure 6B**). The nomograms were constructed by combining all the independent prognostic markers, namely, tumor grades, ALKBH5 mRNA expression levels, IDH mutation status, 1p/19q codeletion status and MGMT promoter status, based on the results of the Cox regression analysis (**Supplementary Figures 6C, D**).

The prognostic efficacy of the factors included in the nomogram were analyzed in the glioma patients from the CGGA_325 and CGGA_693 datasets. The results showed that ALKBH5 mRNA expression levels significantly correlated with the prognosis of glioma patients with different IDH mutations, 1p/19q codeletion, and MGMT promoter status; the high mRNA expression of ALKBH5 negatively correlated with the prognosis of glioma patients in the CGGA_325 and CGGA_693 datasets (**Supplementary Figures 7A–C**). ALKBH5 mRNA expression was associated with the prognosis of the glioma patients in the CGGA_325 and CGGA_693 datasets, but was independent of chemotherapy and/or radiotherapy (**Supplementary Figures 7D, E**).

Overall, the results demonstrated that the ALKBH5 mRNA and protein expression levels were associated with the prognosis of multiple cancers. Moreover, higher ALKBH5 mRNA and protein expression was associated with poorer prognosis of glioma patients.

### Alterations in the *ALKBH5* Gene Are Associated With Development and Progression of Multiple Tumors Including Gliomas

**Figure 3:**
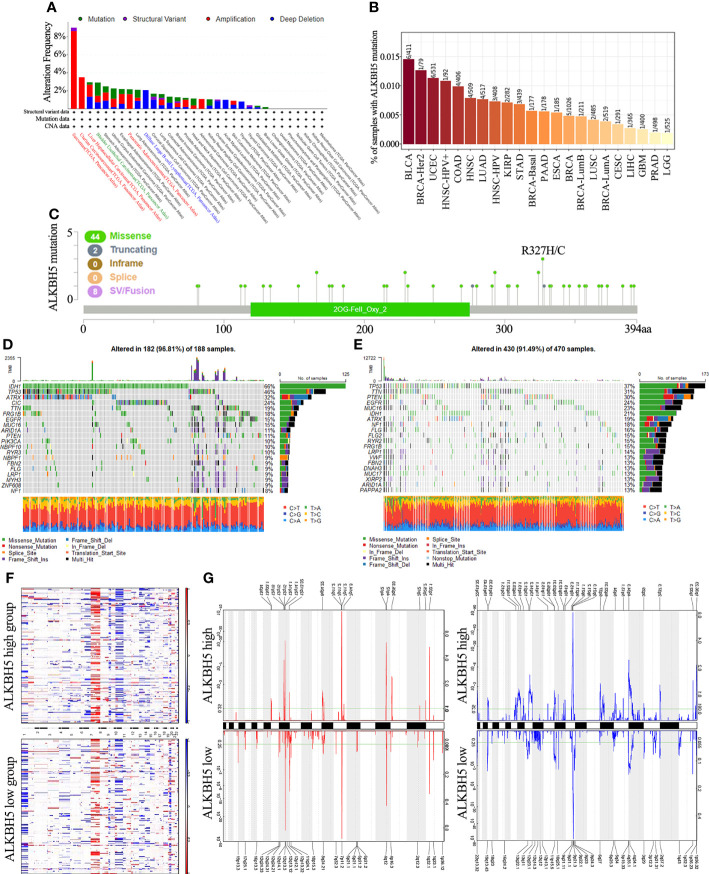
Distinct genomic profiles associated with ALKBH5 expression and integrative analysis of complex cancer genomics and clinical profiles. **(A)** Genetic alteration features (Mutation, Structural Variant, Amplification and Deep Deletion) of ALKBH5 in 32 different tumors were analyzed in TCGA database by the cBioPortal tool. The red label represented the tumors with the top 3 levels of amplification. The green label represented the tumor with the highest mutation level and the blue label represented the tumor with the highest deep deletion level; **(B)** Mutation rates of ALKBH5 gene in various tumors via TIMER portal; **(C)** The mutation sites of ALKBH5 in multiple tumors by the cBioPortal tool. Detection of differential somatic mutations in gliomas, including 25% ALKBH5low group **(D)** and 25% ALKBH5high group **(E)**. Only the top 20 genes with the highest mutation rates were shown; **(F)** The CNAs profile analysis about 25% ALKBH5low group and 25% ALKBH5high group in TCGA dataset via GISTIC2.0; **(G)** Frequency of amplifications and deletions in gliomas with low and high ALKBH5 expression (Blue, deletion; red, amplification).

Genetic alterations such as the mutations, deletions, or amplifications of oncogenes or tumor suppressor genes are associated with growth and progression of several tumors (45). Therefore, we first analyzed different types of alterations including mutations, structural variations, amplifications, and deep deletions in the *ALKBH5* gene in using the TCGA cancer datasets with the cBioPortal portal. The most common genetic alteration in the *ALKBH5* gene were amplifications in the sarcomas (>8%), uterine carcinosarcomas, liver hepatocellular carcinoma and pancreatic adenocarcinomas; mutations (1.3%) in the bladder urothelial carcinomas; and deep deletions (>2%) in the diffuse large B-cell lymphomas (**Figure 3A**). TIMER database analysis showed that BLCA (6/411), BRCA-Her2 (1/79), and UCEC (6/531) were the top 3 cancers with the highest *ALKBH5* gene mutation rates (**Figure 3B**). cBioPortal database analysis showed that missense mutations were the main type of *ALKBH5* gene mutations in cancers (**Figure 3C**).

Next, the relationship between ALKBH5 expression and specific genomic characteristics such as somatic mutations and copy number variations (CNVs) was analyzed in the TCGA glioma dataset. The ALKBH5^high^ group (n = 166) showed high frequency of somatic mutations in the *TP53* (37%), *TTN* (31%), *PTEN* (30%), and *EGFR* (24%) genes and the ALKBH5^low^ group (n = 166) showed high frequency of mutations in the *IDH1* (66%), *TP53* (46%), *ATRX* (32%), and *CIC* (24%) genes (**Figures 3D, E**). The comparison of the CNV profiles in the ALKBH5^low^ (n = 166) and ALKBH5^high^ (n = 166) samples is shown in **Figure 3F**. In the ALKBH5^high^ samples, we observed amplification peaks in the 1q32.1, 3q26.33, 4q12, 7p11.2, 8q24.22, and 12q15 chromosomal regions, and deletions in the 1p36.23, 4q34.3, 9p21.2, 13q14.2 and 13q22.1 chromosomal locations. In the ALKBH5^low^ samples, we observed amplification peaks in the 4q12, 7p11.2, 12q13.3 and 19p13.3 chromosomal locations and frequent deletions in the 4q35.1 and 9p21.3 chromosomal regions (**Figure 3G**).

Overall, these results showed ALKBH5 gene mutations, amplifications, and deletions in multiple tumors. Missense mutations were the most frequent type of ALKBH5 gene mutations in various tumors. Moreover, the glioma tissues showed distinct somatic mutations and CNVs based on the expression levels of ALKBH5. This suggested that alterations in the ALKBH5 gene may regulate the initiation, growth and progression of various tumors, especially gliomas.

### ALKBH5 and Related Genes Regulate Immunity, Immune Signaling, and Metabolism in Gliomas and Other Cancer Types

**Figure 4:**
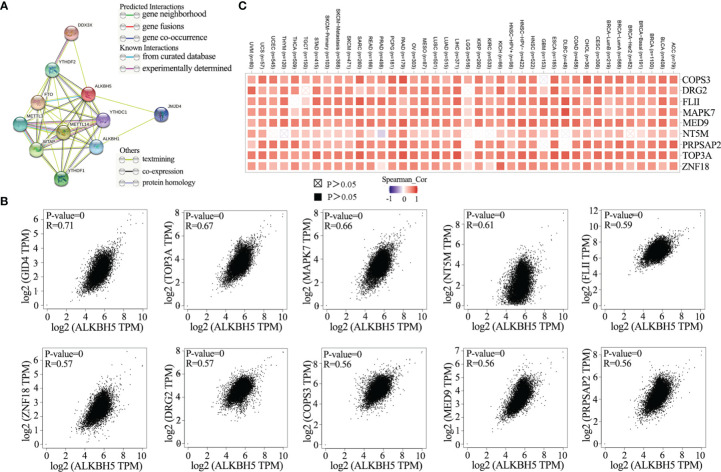
ALKBH5-related genes enrichment analysis in pan-cancers. **(A)** ALKBH5-binding proteins were displayed using the STRING tool (DDX3X, YTHDC1, YTHDF1, YTHDF2, FTO, METTL3, METTL14, WTAP, JMJD4, ALKBH1); **(B)** The top 10 ALKBH5-correlated genes was displayed in various tumors and the correlations between ALKBH5 expression and selected targeting genes were analyzed *via* the GEPIA2 website. These top 10 genes included GID4 (R=0.71), TOP3A (R=0.67), MAPK7 (R=0.66), NT5M (R=0.61), FLII (R=0.59), ZNF18 (R=0.57), DRG2 (R=0.57), COPS3 (R=0.56), MED9 (R=0.56) and PRPSAP2 (R=0.56). The P-value and partial correlation (R) were generated *via* the purity-adjusted Spearman’s rank correlation test; **(C)** The corresponding heatmap data of ALKBH5 and its positively correlated genes in various tumors *via* TIMER2.0.

STRING database analysis identified ten ALKBH5-binding proteins, namely, FTO, METTL3, METTL14, ALKBH1, YTHDC1, YTHDF1, JMJD4, YTHDF2, DDX3X and WTAP as shown in the protein-protein interaction (PPI) network (**Figure 4A**). GEPIA2 database analysis of 33 cancer types showed the following top 10 ALKBH5-related genes: GID4 (R=0.71), TOP3A (R=0.67), MAPK7 (R=0.66), NT5M (R=0.61), FLII (R=0.59), ZNF18 (R=0.57), DRG2 (R=0.57), COPS3 (R=0.56), MED9 (R=0.56) and PRPSAP2 (R=0.56) (all *P*<0.0001; **Figure 4B**). TIMER2 database was then used to confirm the association between ALKBH5 and the top 9 ALKBH5-related genes (GID4 was not annotated in TIMER2 portal) in 40 cancer types (**Figure 4C**).

**Figure 5:**
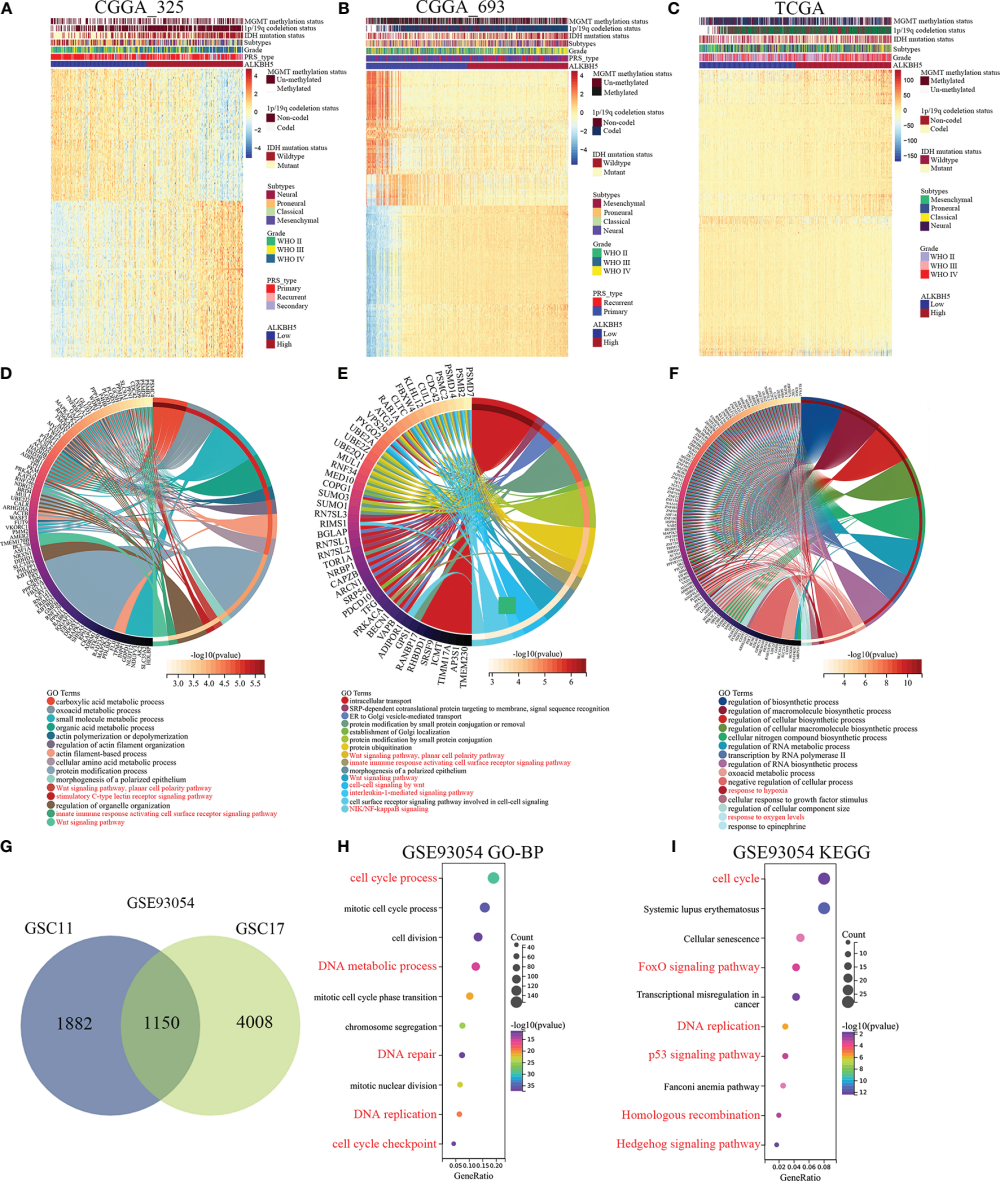
ALKBH5-related genes enrichment analysis in glioma by CGGA_325, CGGA_693 and TCGA datasets. **(A-C)** Heatmap showing the enrichment patterns of the top 200 genes positively (100) or negatively (100) correlated with ALKBH5 expression of glioma in the CGGA_325, CGGA_693 and TCGA databases. **(D-F)** Alterations in different classifications of biological functions in gliomas samples with ALKBH5 expression in the CGGA_325, CGGA_693 and TCGA databases. **(G)** Venn diagram showing the 1150 intersectional genes of differentially expressed genes in GSC11 (3032 differentially expressed genes) and GSC17 (5158 differentially expressed genes) cell lines after ALKBH5 knockdown in GSE93054 dataset. The GO-BP **(H)** and KEGG **(I)** pathway enrichment analyses were applied with the 1150 intersectional genes of differentially expressed genes in GSE93054 dataset. Red label indicated that these GO-BP or KEGG analysis was associated with cell cycle, DNA damage repair, metabolism, or immunity.

Next, in order to further exploring the biological functions of ALKBH5 in glioma. GO-BP and KEGG enrichment analyses of ALKBH5-related genes in glioma was performed. **Figures 5A–C** showed the enrichment patterns of the top 200 genes positively (100 genes) or negatively (100 genes) correlated with ALKBH5 expression in glioma from the CGGA_325, CGGA_693 and TCGA databases. In CGGA_325 and CGGA_693 databases, GO-BP analyses showed wnt signaling pathway, stimulatory C-type lectin receptors signaling pathway, innate immune response activating cell surface receptor signaling pathway, cell-cell signaling by wnt, interleukin-1-mediated signaling pathway and NIK/NF-kappaB signaling (**Figures 5D, E**). In TCGA database, GO-BP analysis showed response to hypoxia and response to oxygen levels (**Figure 5F**). These results showed that ALKBH5 could regulate immunity and metabolism correlated signaling pathways in glioma. In addition, GSE93054 dataset was used to further exploring the correlation between ALKBH5 and immunity and metabolism. **Figure 5G** showed the 1150 intersectional genes of differentially expressed genes in GSC11 (3032 differentially expressed genes) and GSC17 (5158 differentially expressed genes) cell lines after ALKBH5 knockdown in GSE93054 dataset. The GO-BP analysis of these 1150 genes showed the enrichment processes, including cell cycle process, DNA metabolic process, DNA repair, DNA replication and cell cycle checkpoint in **Figure 5H**. And the KEGG enrichment analysis showed that these 1150 genes enriched in the cell cycle, FoxO signaling pathway, DNA repair, p53 signaling pathway, Homologous recombination, Hedgehog signaling pathway and so on (**Figure 5I**).

These results indicated that ALKBH5 expression was associated with cell cycle, DNA damage repair, metabolism, or immunity. Therefore, we then studied the relationship between ALKBH5 and immune in a variety of tumors.

### ALKBH5 Regulates Tumor Infiltration of Immune Cells in Multiple Human Cancers

**Figure 6:**
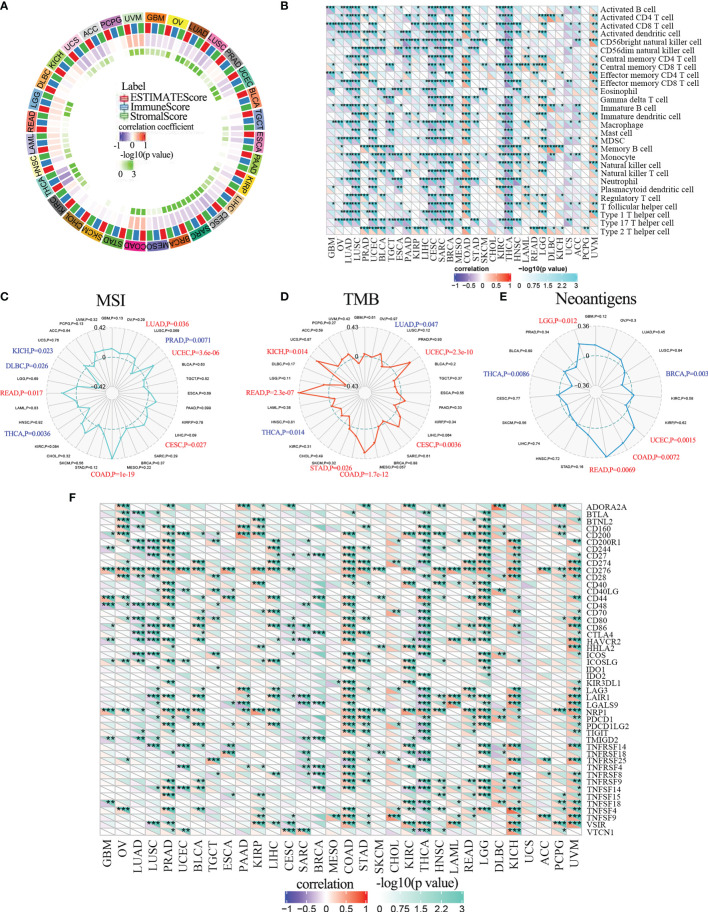
ALKBH5 mRNA expression and immune infiltration. **(A)** The correlations between ESTIMATE scores (ESTIMATE Score, Immune Score, and Stromal Score) and ALKBH5 expression were analyzed in various tumors by Sangerbox portal. **(B)** The relationship between ALKBH5 expression and immune cell infiltration level was analyzed in various tumors by Sangerbox online website. The relationship between ALKBH5 mRNA expression and MSI **(C)**, TMB **(D)**, neoantigen **(E)** and ICP-gene **(F)** in multiple cancers. Red label indicated positive correlation with ALKBH5 expression and blue label indicated negative correlation with ALKBH5 expression. *P<0.05, **P<0.01, ***P<0.001, ****P<0.0001.

The above results suggested that ALKBH5 may regulate the tumor immune microenvironment (TIM) in multiple cancer types, we studied the relationship between ALKBH5 expression levels and the composition of tumor infiltrating immune cells (TIICs) in various tumors. Several studies have shown that TIICs are important components of the tumor microenvironment and regulate tumor initiation, progression, and metastasis (46, 47). Firstly, we evaluated the correlation between ESTIMATE scores (ESTIMATE, immune, and stromal scores) and ALKBH5 expression levels in pan-cancers. Immune score reflects the proportion of infiltrated immune cells in the tumor tissues; stromal score reflects the proportion of stromal cells in the tumor tissues. ESTIMATE score is the sum of immune and stromal scores, and reflects the status of the tumor immune microenvironment and tumor purity. Our results demonstrated negative correlation between ALKBH5 expression and the ESTIMATE, immune, and stromal scores in LUAD, LUSC, UCEC, LIHC, CESC, SARC, BRCA and THCA (**Figure 6A**). This suggested that high ALKBH5 expression was associated with decreased infiltration of immune and stromal cells in several tumors, thereby resulting in high tumor purity. Conversely, ALKBH5 expression showed positive correlation with ESTIMATE, immune, and stromal scores in COAD, LGG, and UVM (**Figure 6A**). **Supplementary Figure 8** showed the positive correlations between ALKBH5 mRNA expression level and ESTIMATE score, Immune score and Stromal score in glioma.

Then, we investigated the relationship between ALKBH5 expression and TIIC levels in various tumors using the Sangerbox database. ALKBH5 expression showed negative correlation with multiple immune cell types in LUAD, LUSC, UCEC, CESC, SARC, BRCA, and THCA, and positive correlation with immune cell types in COAD, LGG and UVM (**Figure 6B**). The immune cell types included activated CD4+ and CD8+ T cells, activated dendritic cells, central memory CD4+ and CD8+ T cells, effector memory CD4+ and CD8+ T cells, gamma delta T cells, immature B cells, macrophages, MDSCs, memory B cells, natural killer cells, natural killer T cells, and regulatory T cells (**Figure 6B**).

### ALKBH5 Regulates Interferon Signaling, Lymphocyte Activation, and Activation of Antigen-Presenting Cells in Glioma

**Figure 7:**
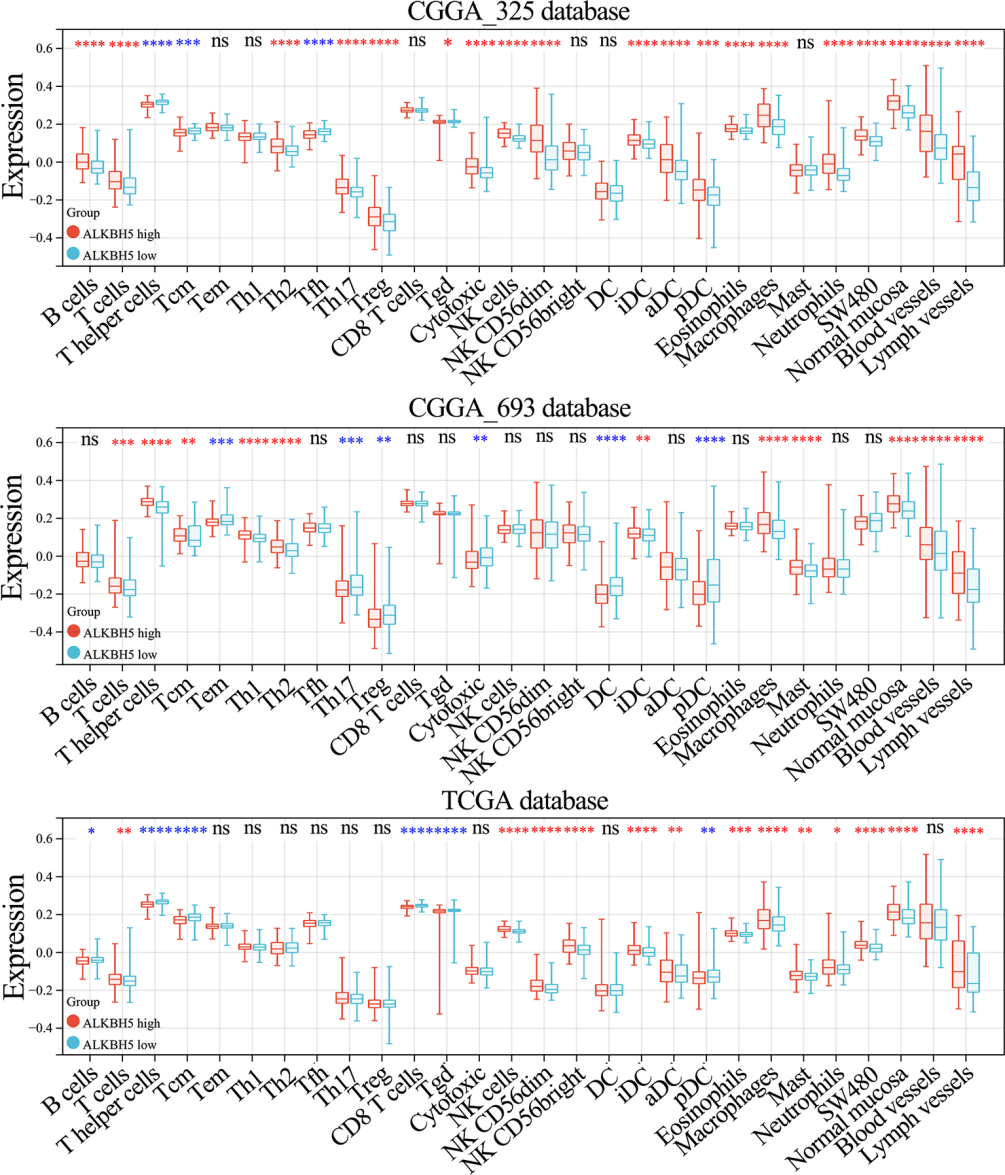
Correlation between ALKBH5 mRNA expression and tumor immune-related cells in glioma. The correlation between ALKBH5 mRNA expression and immune cells types in glioma calculated by xCell algorithm in CGGA_325, CGGA_693 and TCGA datasets. ns (nonsense), *P<0.05, **P<0.01, ***P<0.001, ****P<0.0001.

Since ALKBH5 expression shows positive correlation with tumor infiltration of immune cells in LGG, we further analyzed the relationship between ALKBH5 and tumor infiltrating immune cells in glioma using the ImmuCellAI and xCell databases. ALKBH5 expression levels showed positive correlation with various TIICs in the CGGA_325, CGGA_693, and TCGA glioma datasets; moreover, ALKBH5 expression correlated with the proportions of immune and stromal cells in the gliomas (**Figure 7** and **Supplementary Figure 9**). This suggested that ALKBH5 expression modulated the recruitment of immune cells into the glioma and may affect the sensitivity of glioma patients to immunotherapy.

We then further investigated the effects of ALKBH5 expression on the tumor immune microenvironment (TIM) of glioma by screening seven metagenes, namely, *HCK, IgG, Interferon, LCK, MHC-I, MHC-II*, and *STAT1*, which reflect the status of inflammation and immune responses (48, 49). Our results showed higher enrichment scores for the lymphocyte-specific kinase (*LCK*), major histocompatibility complex I (*MHC-I*), major histocompatibility complex II (*MHC-II*) and signal transducer and activator of transcription 1 (*STAT1*) metagenes in glioma patients with high ALKBH5 expression levels from the CGGA_325, CGGA_693, and TCGA datasets (**Supplementary Figure 10**). This suggested that ALKBH5 expression regulated interferon signaling, lymphocyte activation, and activation of antigen-presenting cells in the gliomas.

### ALKBH5 Correlates With Immune Checkpoint Blockade Proteins and Is a Potential Biomarker of Tumor Immunotherapeutic Response in Multiple Tumors

Antitumor immunity is a strong predictor of the efficacy of tumor immunotherapy and correlates with microsatellite instability (MSI), tumor mutation burden (TMB), and neoantigens in the tumor microenvironment (50). Tumors with high MSI (MSI-H) and TMB respond favorably to immune checkpoint inhibition therapies (51). Neoantigens are mutated antigens that are specifically expressed by the tumor tissues and are potential targets for T cell-based tumor immunotherapy (52). Therefore, we investigated the relationship between ALKBH5 expression levels and MSI, TMB or neoantigens to determine if ALKBH5 was a predictor of immunotherapeutic responses in multiple cancer types. ALKBH5 expression showed positive correlation with MSI in LUAD (*P*=0.038), UCEC (*P*=3.6e-06), CESC (*P*=0.027), COAD (*P*=1e-19) and READ (*P*=0.017), and negative association with MSI in PRAD (*P*=0.0071), THCA (*P*=0.0036), DLBC (*P*=0.026) and KICH (*P*=0.023) (**Figure 6C**). Furthermore, ALKBH5 expression showed positive relationship with TMB in UCEC (*P*=2.3e-10), CESC (*P*=0.0036), COAD (*P*=1.7e-12), STAD (*P*=0.026), READ (*P*=2.3E-07) and KICH (*P*=0.014), and negative association with TMB in LUAD (*P*=0.047) and THCA (*P*=0.014) (**Figure 6D**). ALKBH5 expression also showed positive correlation with neoantigens in UCEC (*P*=0.0015), COAD (*P*=0.0072), READ (*P*=0.0069) and LGG (*P*=0.012), and negative association with neoantigens in BRCA (*P*=0.003) and THCA (*P*=0.0086) (**Figure 6E**).

The immune checkpoint (ICP) blockade proteins are the most promising targets of cancer immunotherapeutic treatments because they regulate the infiltration of immune cells into the tumor microenvironment (48). Therefore, we analyzed the relationship between expression levels of ICP genes and ALKBH5 in multiple cancer types. ALKBH5 expression showed a positive correlation with ICP genes in UVM, KICH, LGG and COAD (**Figure 6F**). ALKBH5 expression showed positive correlation with 34 out of 47 ICP genes in COAD and 32 out of 47 ICP genes in UVM (**Figure 6F**). These results suggested that ALKBH5 affected the sensitivity of UVM, KICH, LGG and COAD to the immune checkpoint inhibitor therapies. In THCA, ALKBH5 expression showed negative correlation with the ICP genes (**Figure 6F**). This suggested that THCA patients with high ALKBH5 expression may respond poorly to immunotherapies targeting ICP genes. Next, we analyzed the correlation between ALKBH5 and various ICP receptors and ligands in glioma patients from the CGGA_325, CGGA_693, and TCGA datasets (49). ALKBH5 expression showed positive association with ICP receptors such as TNFRSF14, CD40, CD96 and CD200R1, and ICP ligands such as PDCD1LG2, CD70, TNFSF14, ICOSLG and CD274 in glioma tissues (**Supplementary Figure 11**). Therefore, we hypothesized that high ALKBH5 expression altered the immune microenvironment in the glioma tissues by modulating the expression levels of ICP receptors and ligands such as TNFRSF14, CD40, CD96, PDCD1LG2, CD70 and TNFSF14. This suggested that ALKBH5 mediated the activation of ICP genes and was an ideal target for immunotherapy of glioma patients.

Overall, our results suggested that ALKHB5 expression levels modulated the sensitivity of several tumors to immunotherapy. Therefore, ALKBH5 is a potential immunotherapy biomarker and predictor of tumor immunotherapeutic response.

### Construction of the Upstream lncRNA-miRNA Regulatory Network That Regulates ALKBH5 Expression Levels in Gliomas and Other Tumors

**Figure 8:**
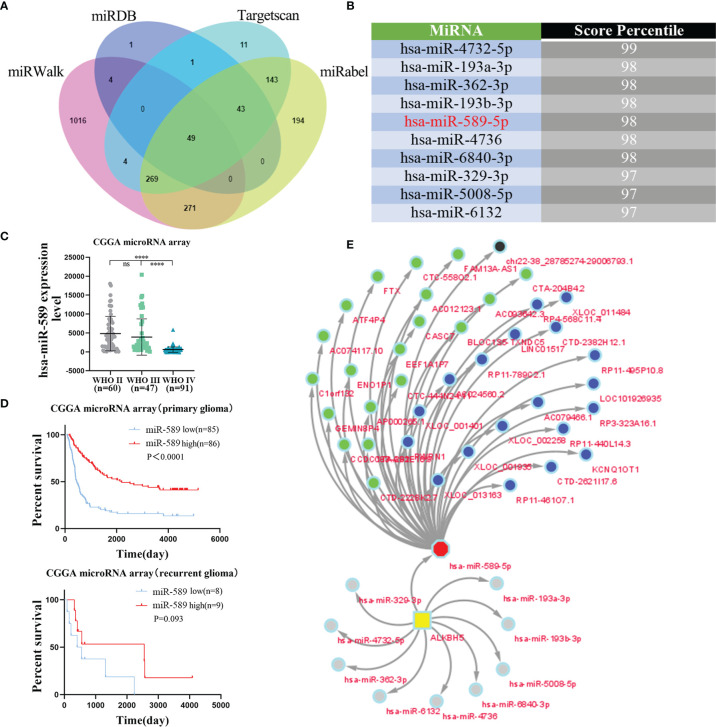
The lncRNA-miRNA-ALKBH5 regulatory network constructed. **(A)** The upstream miRNAs of ALKBH5 were predicted by miRWalk, miRDB, Targetscan and miRabel databases and the intersection was taken (49 intersection miRNAs). **(B)** The top 10 miRNAs targeting ALKBH5 were displayed in Targetscan database. **(C)**The relationship between hsa-miR-589 expression and grade in glioma from CGGA microRNA array dataset. **(D)** The relationship between hsa-miR-589 expression and prognosis in glioma patients from CGGA microRNA array dataset. **(E)**The lncRNA-miRNA-ALKBH5 regulatory network was constructed by Cytoscape. ns (nonsense), ****P<0.0001.

In recent years, several studies have shown that long non-coding RNAs (lncRNAs) play a significant role in tumorigenesis by regulating the expression of the downstream mRNAs through sequestering of their target miRNAs (53, 54). Therefore, we investigated the lncRNA-miRNA network that may regulate ALKBH5 expression in various tumors. First, we screened the miRWalk, miRDB, Targetscan, and miRabel databases and identified 49 miRNAs that potentially target the ALKBH5 mRNAs (**Figure 8A**). The top 10 ALKBH5 mRNA-targeting miRNAs were hsa-miR-4732-5p, hsa-miR-193a-3p, hsa-miR-362-3p, hsa-miR-193b-3p, hsa-miR-589-5p, hsa-miR-4736, hsa-miR-6840-3p, hsa-miR-329-3p, hsa-miR-5008-5p and hsa-miR-6132 (**Figure 8B**).

Among these 10 ALKBH5 mRNA-targeting miRNAs, 5 miRNAs (hsa-miR-193a-3p, hsa-miR-362-3p, hsa-miR-193b-3p, hsa-miR-589-5p and hsa-miR-329-3p) were found in the CGGA database (**Figures 8C, D** and **Supplementary Figure 12**). Bioinformatics analysis showed that the expression levels of hsa-miR-193a-3p (**Supplementary Figure 12A**), hsa-miR-362-3p (**Supplementary Figure 12C**), and hsa-miR-193b-3p (**Supplementary Figure 12E**) were positively associated with glioma grades, whereas, the expression levels of hsa-miR-589-5p (**Figure 8C**) and hsa-miR-329-3p (**Supplementary Figure 12G**) showed negative correlation with glioma grades. Kaplan-Meier survival curve analysis of glioma patients in the CGGA database showed that hsa-miR-589-5p was associated with the prognosis of primary glioma patients (**Figure 8D**). The expression levels of hsa-miR-329-3p were not associated with the prognosis of glioma patients (**Supplementary Figure 12H**). These results suggested that hsa-miR-589-5p potentially targeted ALKBH5 expression in the gliomas.

Next, we identified 769 predicted lncRNAs and 73 validated lncRNAs that may target hsa-miR-589-5p using the LncBase database. The top 20 predicted lncRNAs and top 20 validated lncRNAs were used to construct a lncRNA-miRNA-ALKBH5 regulatory network using the cytoscape software (**Figure 8E**). These results demonstrated the upstream lncRNA-miRNA regulatory network that may regulate the aberrant expression of ALKBH5 in the gliomas.

### ALKBH5 Promotes *In Vitro* and *In Vivo* Proliferation, Migration, and Invasion of Glioma Cells and Affect the Recruitment of M2 Macrophages

**Figure 9:**
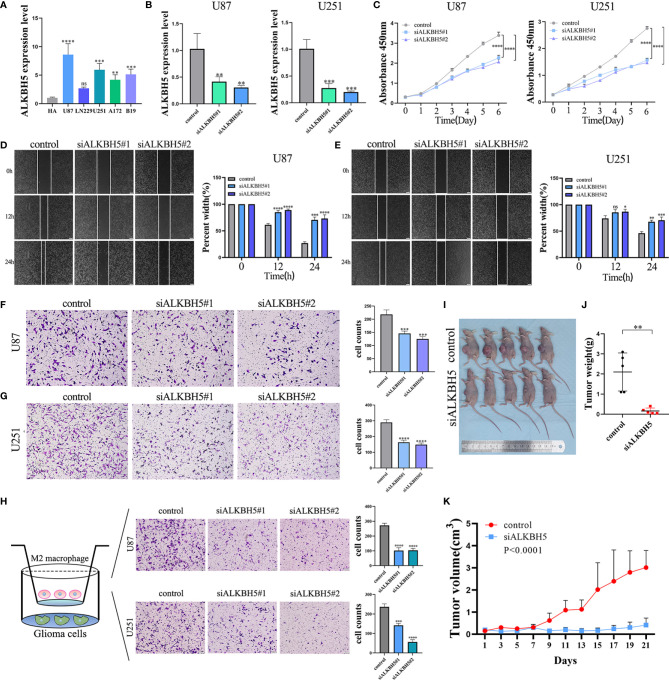
The biological functions of ALKBH5 in glioma. **(A)** The expression levels of ALKBH5 were verified in multi-cell lines, including human astrocyte (HA), U87, LN229, U251, A172 and B19 cell lines. **(B)** Verification of knockdown efficiency of ALKBH5 in U87 and U251 cell lines. The biological functions of ALKBH5 on glioma cell lines were verified by CCK-8 **(C)**, wound healing **(D, E)** and Transwell **(F, G)** experiments. Xenograft mouse models were generated by subcutaneous injection of U87 cells into nude mice. Seven days after implanted, mice were treated with control siRNA (n=8) or ALKBH5 siRNA (n=8). **(H)** Infiltration of M2 macrophage in control (left), siALKBH5#1 (middle), siALKBH5#2 (right) of U87 and U251 cells. Representative image of subcutaneous tumors **(I)**, tumor weight **(J)** and tumor growth curve **(K)**, in si-ALKBH5 treated or control siRNA treated xenograft models with U87 cells. ns (nonsense), *P<0.05, **P<0.01, ***P<0.001, ****P<0.0001.

Next, we analyzed ALKBH5 expression levels in various glioma cell lines by qRT-PCR. ALKBH5 mRNA levels were significantly higher in the U87-MG and U251-MG cell lines compared to the other glioma cell lines and were consistent with the results from the CCLE database (**Figure 9A** and **Supplementary Figure 13**). We then used specific ALKBH5-targeting siRNAs to knockdown the expression levels of ALKBH5 in the U87-MG and U251-MG cells (**Figure 9B**). And ALKBH5 was overexpressed by using the ALKBH5 overexpression plasmid (**Supplementary Figure 14A**). CCK-8 assay, Wound healing assay and Transwell assay results showed that ALKBH5 knockdown suppressed the cell proliferation, migration and invasion of U87-MG and U251-MG and ALKBH5 overexpression promoted the cell proliferation, migration and invasion of LN229 *in vitro* (**Figures 9C-G** and **Supplementary Figures 14B–D**). Moreover, THP-1 cells were differentiated into M2 macrophage on the basis of classical inducing methods (**Supplementary Figure 15**) (55–57). Silencing of ALKBH5 in U87 and U251 cells significantly reduced the infiltration of M2 macrophages (**Figure 9H**), consisting with the results of our previous analysis (**Figure 7** and **Supplementary Figure 9**). Furthermore, tumor xenograft experiments showed that ALKBH5 knockdown suppressed the *in vivo* growth of glioma cells (**Figures 9I–K**). These results showed that ALKBH5 promoted *in vitro* and *in vivo* proliferation, migration, and invasion of glioma cells.

## Discussion

N^6^-methyladenosine (m^6^A) modification is the most common chemical modification in messenger RNAs (mRNAs), and non-coding RNAs (ncRNAs) (58). The reversible N6-methylation of adenosine is a dynamic post-transcriptional modification process involving three protein complexes, namely, m^6^A readers, m^6^A writers, and m^6^A erasers (59). Several studies have shown that aberrant m^6^A modification regulates the development, growth, and progression of various tumors (1).

ALKBH5 is a m^6^A demethylase that is associated with the development and progression of multiple cancer types (60), such as glioblastoma, ovarian cancer, lung cancer, liver cancer, pancreatic cancer and osteosarcoma. However, pan-cancer analysis of ALKBH5 has not been investigated. Therefore, we investigated the functional role of ALKBH5 in multiple tumors, especially gliomas. The analysis included the effects of ALKBH5 RNA expression levels on prognosis, as well as the genetic alterations in the *ALKBH5* gene, GO and KEGG analyses of ALKBH5-related genes, and tumor immunity. We also investigated the upstream lncRNA-miRNA network that regulates ALKBH5 expression in gliomas and its effects on glioma progression.

In this study, we first performed comprehensive bioinformatics analysis to determine the functional role of ALKBH5 in multiple cancers using the patient data from various databases. The results showed that ALKBH5 mRNA and protein levels were upregulated compared to the corresponding normal tissues in multiple cancers including glioma. Our analysis was confirmed by the previous studies. ALKBH5 mRNA was increased in epithelial ovarian cancer tissue as compared to the normal ovarian tissues and ALKBH5 silencing could inhibit the proliferation and invasion of epithelial ovarian cancer cells (16). Xu et al. found that ALKBH5 protein expression was increased in colon cancer and could promote colon cancer progression by decreasing methylation of the lncRNA NEAT1 (61). Gu et al. discovered that the mRNA and protein expressions of ALKBH5 were overexpressed in endometrial cancer cells and promoted proliferation and invasion of endometrial cancer *via* erasing IGF1R m^6^A-modifications (62). For glioma, Ji et al. found that the mRNA and protein expressions of ALKBH5 were upregulated in glioma and promoted the glioma cells proliferation by increasing the mRNA stability of G6PD (63). ALKBH5 expression was associated with tumor grades, subtypes, and new WHO tumor types (IDH mut+codel, IDH mut+non-codel and IDH wild) in glioma. The pan-cancer analysis showed correlation between ALKBH5 mRNA expression and the prognosis of cancer patients. Analysis of 100 glioma cases discovered that glioma patients with high ALKBH5 expression levels had shorter OS compared with patients with low ALKBH5 expression level. IDH mutations, considered as truncal events, are occurred in the vast majority of low-grade gliomas (LGG) and secondary GBM (64). The 2016 WHO Classification of CNS tumors included several tumor-specific molecular alterations in the classification and diagnosis of gliomas. The 1p/19q co-deletion status were included in the oligodendroglioma stratification. Multiple studies have shown that 1p/19q co-deletion had significant prognostic value in the WHO oligodendrocytoma subgroup (49). In our studies, high ALKBH5 expression was associated with clinical features such as IDH wildtype status, 1p/19q non-codeleted status, and demethylation of MGMT, all of which were related to poor prognosis in the glioma patients. Cox regression analysis showed that ALKBH5 was an independent prognostic predictor in glioma patients. Recent research showed that ALKBH5 mRNA and protein expression were upregulated in glioblastoma stem-like cells (GSCs) and ALKBH5 could maintain the tumorigenicity of GSCs by maintaining FOXM1 expression (15). And Guo et al. found that lncRNA SOX2OT could enhance SOX2 expression through ALKBH5-mediated epigenetic regulation in glioblastoma, thereby promoting temozolomide resistance (65). Moreover, Catherine Seva et al. reported that ALKBH5 promoted radio-resistance and invasion capability of glioblastoma stem cells (GBMSCs) (66). These findings demonstrated that high ALKBH5 expression was associated with unfavorable survival outcomes in gliomas. Overall, our analysis showed that ALKBH5 was a potential prognostic biomarker in various cancers, especially in glioma.

Somatic mutations in critical genes transform normal cells into cancer cells (67). Moreover, somatic mutations in the cancer cells contribute significantly to immune evasion and poor responses to therapies (68). We identified amplifications, mutations, and deep deletions as the most frequent alterations in the *ALKBH5* gene in several cancer types. In this study, gliomas with low ALKBH5 expression showed higher proportion (66%) of IDH1 mutations compared to those with high ALKBH5 expression (21%). Previous reports showed that IDH mutations were more abundant in LGG, and the survival rate of LGG patients was higher than those with HGG (69). The low ALKBH5 expressing glioma patients (46%) also showed increased frequency of mutations in *TP53* compared to the high ALKBH5 expressing group (37%). TP53 is a well-known tumor suppressor gene that inhibits GBM progression. The high ALKBH5-expressing glioma patients showed higher EGFR mutational rate compared to those with low ALKBH5 expression (24% vs. 15%). Previous studies showed that EGFR activation in patients with GBM was associated with worse survival outcomes (70). Therefore, ALKBH5 is a potential prognostic biomarker for patients with glioma and multiple cancer types.

In order to explore the biological functions of ALKBH5, we applied GO and KEGG analysis of ALKBH5 related genes. GO and KEGG pathway enrichment analysis showed that high ALKBH5 expression correlated with the activation of metabolic and immune signaling pathways in various tumors. In glioma, ALKBH5 could affect immunity, metabolism, cell cycle, DNA damage repair *via* CGGA_325, CGGA_693, TCGA and GSE93054 datasets. We focused on the effect of ALKBH5 on immunity, for the significant role of immune system in preventing and fighting tumors. In recent years, cancer immunotherapy, restoring the immune system’s ability to recognize and destroy tumor cells, has shown significant clinical results, but only for a small number of tumors (64). And the tumor-infiltrating immune cells play a significant role in the tumor microenvironment (TME), which provides a favorable microenvironment for the survival of cancer cells, and is associated with regulation of tumor immunosurveillance and therapeutic response of tumors (71, 72). ESTIMATE analysis showed that high expression of ALKBH5 correlated significantly with the infiltration of immune cells and stromal cells in multiple cancers. These results suggested that ALKBH5 inhibited the infiltration of immune cells into tumors, thereby enabling the tumor cells to evade the immune system. Tariq M Rana et al. found that ALKBH5 could regulate the expression of Mct4/Slc16a3 and lactate content in tumor microenvironment, as well as the composition of tumor infiltrating Treg and bone marrow derived inhibitory cells, thereby inhibiting immune cells accumulation in tumor microenvironment in some tumors, which was consistent with our analysis (23).

In recent years, immunotherapy has been widely applied in tumors, but many solid tumors showed poor immunotherapy effect due to their special immunosuppressive microenvironment (49). As an immunologically “cold tumor”, glioma is considered to be highly resistant to immunotherapy. However, our research found that ALKBH5 expression was positively correlated with the infiltration of immune cells into the tumors in glioma and may affect the sensitivity of glioma patients to immunotherapy. Wu et al. found that ALKBH5 promoted the generation of immunosuppressive tumor microenvironment *via* hypoxia-induced paraspeckle assembly and IL8 secretion (73). These results suggested that ALKBH5 could be acted as a potential therapeutic target for glioma and as a novel marker to predict prognosis of glioma patients (74). In addition, analysis of the seven inflammation and immune related clusters suggested that high ALKBH5 expression activated the antigen-presenting cells and lymphocytes, and enhanced interferon signaling in the glioma tissues. Overall, these data suggested that glioma patients with high ALKBH5 expression may benefit from immunotherapy. This study provides theoretical basis for the application of immunotherapy in patients with glioma (49).

Recent studies have shown that tumors with high MSI (MSI-H), TMB or neoantigens show better response to immunotherapy (75). ICP proteins play a significant role in the tumor-infiltration of immune cells and immunotherapy (76). Therefore, ICP gene expression is used to select tumor patients that may benefit from immune checkpoint blockage (ICB) therapy (77). Our results showed that the expression of ALKBH5 was closely related to MSI, TMB, and neoantigens in various tumors. ALKBH5 expression correlated with the expression of multiple ICP genes in multiple cancers. This suggested that ALKBH5 regulated tumor immunity in several cancer types. Therefore, ALKBH5 may be a potential predictive biomarker of immunotherapeutic responses in patients with malignant tumors. Finally, we used Targetscan, miWalk, miRDB, miRabel and other websites to identify ALKBH5-targeting miRNAs and lncRNAs and constructed a lncRNA-miRNA-ALKBH5 regulatory network that regulates the abnormal expression of ALKBH5 in various cancers. Furthermore, *in vivo* and *in vitro* experiments with glioma cells showed that ALKBH5 acted as an oncogene and promoted the growth, migration, and invasiveness of the glioma cells. And ALKBH5 knockdown could reduce the infiltration of M2 macrophage in U87 and U251 cells.

In summary, our results suggested that ALKBH5 was aberrantly expressed in various cancers and significantly correlated with the prognosis of cancer patients. *ALKBH5* gene alterations including mutations, duplications, and amplifications were identified in a wide variety of cancer types. ALKBH5 expression showed significant association with the infiltration of immune cells into the tumor microenvironment and immunotherapeutic response in multiple cancers. Therefore, ALKBH5 is a potential immunotherapeutic biomarker for selecting tumor patients that may benefit from immune checkpoint blockade (ICB) therapy in glioma and other cancers. *In vitro* and *in vivo* experiments confirmed that ALKBH5 functioned as an oncogene in gliomas. Our study suggested that ALKBH5 was a promising prognostic biomarker as well as a potential predictor of sensitivity to immunotherapy in several malignant tumors and glioma.

## List of abbreviations

The information is shown in **Supplementary Table 1**.

## Data Availability Statement

The original contributions presented in the study are included in the article/**Supplementary Material**. Further inquiries can be directed to the corresponding authors.

## Ethics Statement

The animal study was reviewed and approved by Ethics Committee of the Tianjin Neurological Institute and Hospital (Tianjin Medical University General Hospital).

## Author Contributions

CW, BW and DP contributed equally to this study and made substantial contributions to conception and design, acquisition of data, and analysis and interpretation of data. LL and ZZ supplied the RNA-seq data and clinical information of 100 glioma patients. XZ, LL, ZL, YH and XD performed the experiments and were involved in drafting the article. HL, SL and SZ were mainly responsible for editing the data and revised the article critically for important intellectual content. LH, ZZ and JZ had given final approval of the version to be published. All authors read and approved the final manuscript.

## Funding

The project was supported by the grants (Nos. 81773187, 81702465) from National Nature Science Foundation of China and by Tianjin high school program for young and middle-aged talents backbone and Tianjin young medical talents program.

## Conflict of Interest

The authors declare that the research was conducted in the absence of any commercial or financial relationships that could be construed as a potential conflict of interest.

## Publisher’s Note

All claims expressed in this article are solely those of the authors and do not necessarily represent those of their affiliated organizations, or those of the publisher, the editors and the reviewers. Any product that may be evaluated in this article, or claim that may be made by its manufacturer, is not guaranteed or endorsed by the publisher.

## Supplementary Material

Supplementary Figure 1 Flow chart of bioinformatics analyses.

Supplementary Figure 2 ALKBH5 expression in different IDH status, 1p/19q status and MGMT promotor status in glioma. The expression level of ALKBH5 in different IDH status **(A)**, 1p/19q status **(B)** and MGMT promotor status **(C)** of gliomas in CGGA_325, CGGA_693 and TCGA databases. *P<0.05, **P<0.01, ***P<0.001, ****P<0.0001.

Supplementary Figure 3 ALKBH5 protein expression, location and conservation. **(A)** ALKBH5 protein expression in 44 normal tissues via THPA portal; **(B)** Localization of ALKBH5 protein in cells was displayed via THPA portal; **(C)** Conservation of ALKBH5 protein among different species through NCBI; **(D)** ALKBH5 protein expression in multiple tumors compared with adjacent normal tissues in TCGA database, including ovarian cancer (P=0.0178), breast cancer (P<0.0001), clear cell RCC (P<0.0001), lung adenocarcinoma (P<0.0001) and UCEC (P=0.0126).

Supplementary Figure 4 Correlation between ALKBH5 mRNA expression and prognosis of tumor patients. The relationships between ALKBH5 expression level and OS **(A)** and DSS **(B)** of various cancers by SangerBox (P<0.05). The Kaplan-Meier plotter was applied to survival analysis by ALKBH5 mRNA expression level in ovarian cancer **(C)**, gastric cancer **(D)**, lung cancer **(E)** and liver cancer **(F)**, including overall survival (OS), progression free survival (PFS), first progression (FP), post progression survival (PPS) and relapse free survival (RFS).

Supplementary Figure 5 The relationships between ALKBH5 expression and prognosis of glioma patients. Kaplan-Meier analysis of ALKBH5 expression in the overall survival of patients with primary gliomas **(A)**, recurrent glioma, low grade glioma (LGG), and glioblastoma (GBM) in CGGA_325 **(A)**, CGGA_693 **(B)** and TCGA **(C)** databases.

Supplementary Figure 6 Univariate and multivariate Cox analyses of ALKBH5 and clinical features in glioma. Univariate and multivariate Cox analyses of ALKBH5 and clinical features of glioma in CGGA_325 combined with CGGA_693 **(A)** and TCGA **(B)** databases. The nomogram model of ALKBH5 and clinical features in CGGA_325 combined with CGGA_693 **(C)** and TCGA datasets **(D)**. *P<0.05, **P<0.01, ***P<0.001, ****P<0.0001.

Supplementary Figure 7 Prognosis analyses of glioma patients. Kaplan-Meier analyses of overall survival among four patient groups stratified by the ALKBH5 mRNA level and IDH status **(A)**, 1p/19q status **(B)**, MGMT promotor status **(C)**, radiotherapy **(D)**, and chemotherapy **(E)** in CGGA_325 and CGGA_693 datasets. *P<0.05, **P<0.01, ***P<0.001, ****P<0.0001.

Supplementary Figure 8 Correlation between ALKBH5 mRNA expression and ESTIMATE Score in glioma. The correlations between ALKBH5 mRNA expression level and ESTIMATE Score (P=5.5e-3, R=0.13) (A), Immune Score (P=0.02, R=0.09) (B) and Stromal Score (P=9.0e-4, R=0.13) (C) of glioma in TCGA dataset.

Supplementary Figure 9 Relationship between ALKBH5 expression and immune cell infiltration in glioma. Comparison of immune cell infiltration between ALKBH5 high expression and ALKBH5 low expression groups in glioma by CGGA_325 **(A)**, CGGA_693 **(B)** and TCGA **(C)** datasets via ImmuCellAl algorithm. Red asterisk represents the positive correlation between ALKBH5 expression and the level of immune cell infiltration, blue asterisk represents the negative correlation between ALKBH5 expression and the level of immune cell infiltration. *P<0.05, **P<0.01, ***P<0.001, ****P<0.0001.

Supplementary Figure 10 Correlation analysis between ALKBH5 and immune and inflammatory-related metagenes in glioma. Enrichment scores of seven immune and inflammatory-related metagenes in glioma patients with different ALKBH5 mRNA expression level status in CGGA_325 **(A)**, CGGA_693 **(B)** and TCGA **(C)** datasets. *P<0.05, **P<0.01, ***P<0.001, ****P<0.0001.

Supplementary Figure 11 Correlation between ALKBH5 and immune checkpoint (ICP) genes in glioma. The correlation between ALKBH5 expression and ICP genes (receptors and ligands) of glioma in CGGA_325 **(A)**, CGGA_693 **(B)** and TCGA **(C)** datasets. The red dots represent the positive correlation between ALKBH5 and ICP genes, while the green dots represent the negative correlation between ALKBH5 and ICP genes.

Supplementary Figure 12 The relationships between the expressions of miRNAs and grade and prognosis in glioma. The relationships between the expressions of has-miR-193a-3p **(A, B)**, has-miR-362-3p **(C, D)**, has-miR-193b **(E, F)** and miR-329 **(G, H)** and grades or prognosis of primary and recurrent glioma in CGGA microRNA array database. *P<0.05, **P<0.01, ***P<0.001, ****P<0.0001.

Supplementary Figure 13 The abundance of ALKBH5 mRNA expression in a variety of glioma cell lines by CCLE database. Cell lines labeled in red (U87-MG, U251-MG, A172 and LN229) were used to detect the relative expression level of ALKBH5 in this study *via* qPCR.

Supplementary Figure 14 The biological functions of ALKBH5 in glioma. **(A)** Verification of overexpression efficiency of ALKBH5 via RT-qPCR in LN229 cell lines. The biological functions of ALKBH5 on LN229 cell lines were verified by CCK-8 **(B)**, wound healing **(C)** and Transwell **(D)** experiments. *P<0.05, **P<0.01, ***P<0.001, ****P<0.0001.

Supplementary Figure 15 Morphological changes of THP1 cells induced by PMA, IL-4 and IL-13. Morphological changes of THP1 cells were observed 24 h after PMA induction and 24h/48 h after IL-4 and IL-13 induction.
