# Corrigendum: Pan-Cancer Analysis Shows That ALKBH5 Is a Potential Prognostic and Immunotherapeutic Biomarker for Multiple Cancer Types Including Gliomas

**DOI:** 10.3389/fimmu.2022.944740

**Published:** 2022-06-23

**Authors:** Cheng Wei, Bo Wang, Dazhao Peng, Xiaoyang Zhang, Zesheng Li, Lin Luo, Yingjie He, Hao Liang, Xuezhi Du, Shenghui Li, Shu Zhang, Zhenyu Zhang, Lei Han, Jianning Zhang

**Affiliations:** ^1^ Tianjin Neurological Institute, Key Laboratory of Post-Neuroinjury Neuro-repair and Regeneration in Central Nervous System, Ministry of Education and Tianjin City, Tianjin Medical University General Hospital, Tianjin, China; ^2^ Department of Neurosurgery, The First Affiliated Hospital of Zhengzhou University, Zhengzhou, China; ^3^ Department of Hepatopancreatobiliary Surgery, The Second Hospital of Tianjin Medical University, Tianjin, China

**Keywords:** ALKBH5, pan-cancer, prognosis, immune, glioma, lncRNA-miRNA-ALKBH5 network

In the original article, there was a mistake in Figure 2 and Figure 3 as published. The order of Figure 2 and Figure 3 was reversed. The corrected **Figure 2** and **Figure 3** appear below.

**Figure 2:**
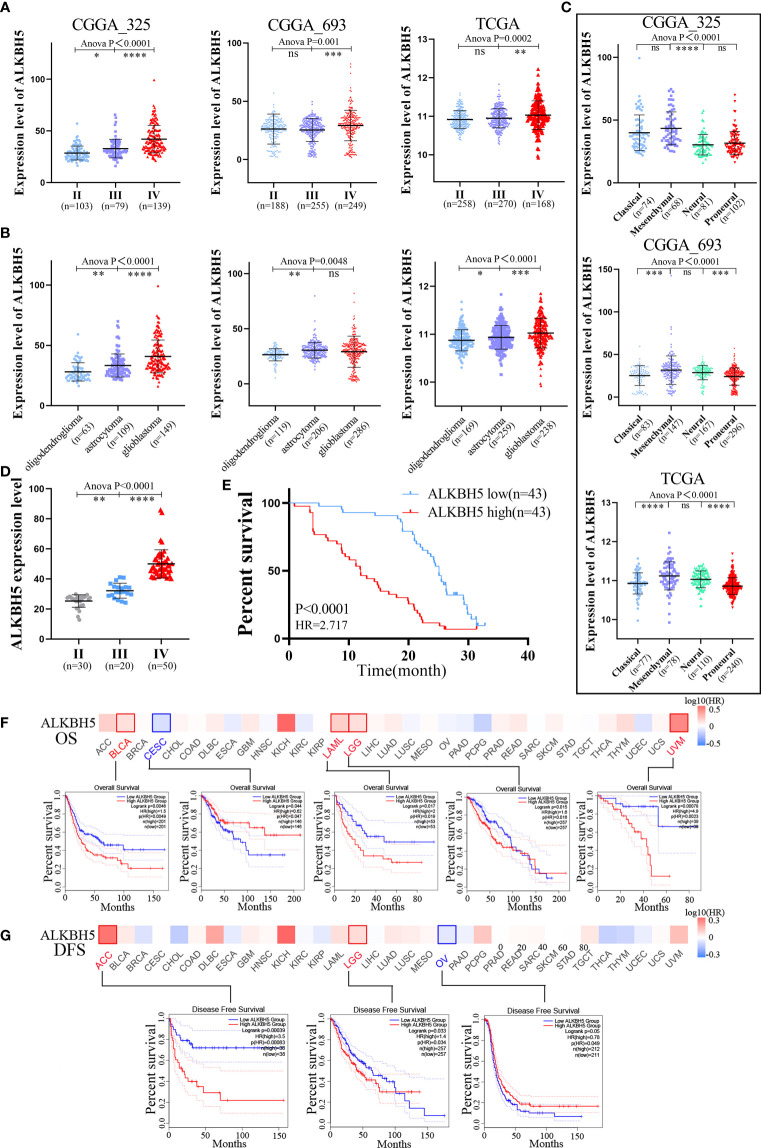
Clinical and molecular characteristics of ALKBH5 in pan-cancer, including gliomas. **(A)** The correlation between ALKBH5 expression and glioma WHO grade (II, II and IV) in CGGA_325, CGGA_693 and TCGA datasets; **(B)** The correlation between ALKBH5 expression and glioma three types (oligodendroglioma, astrocytoma and glioblastoma) in CGGA_325, CGGA_693 and TCGA datasets; **(C)** The relationship between ALKBH5 expression and glioma subtypes (classical, mesenchymal, neural, and proneural) in CGGA_325, CGGA_693 and TCGA datasets; **(D)** The correlation between ALKBH5 expression and grades in 100 glioma samples, including grade II (n=30), grade III(n=20) and grade IV (n=50) samples; **(E)** Overall survival (OS) of different ALKBH5 expression level in 100 glioma samples. The GEPIA2 tool was used to perform OS **(F)** and disease-free survival (DFS) **(G)** analyses of different tumors in TCGA by ALKBH5 expression. Red label indicated positively correlated, blue label indicated negatively correlated. *P<0.05, **P<0.01, ***P<0.001, ****P<0.0001.

**Figure 3:**
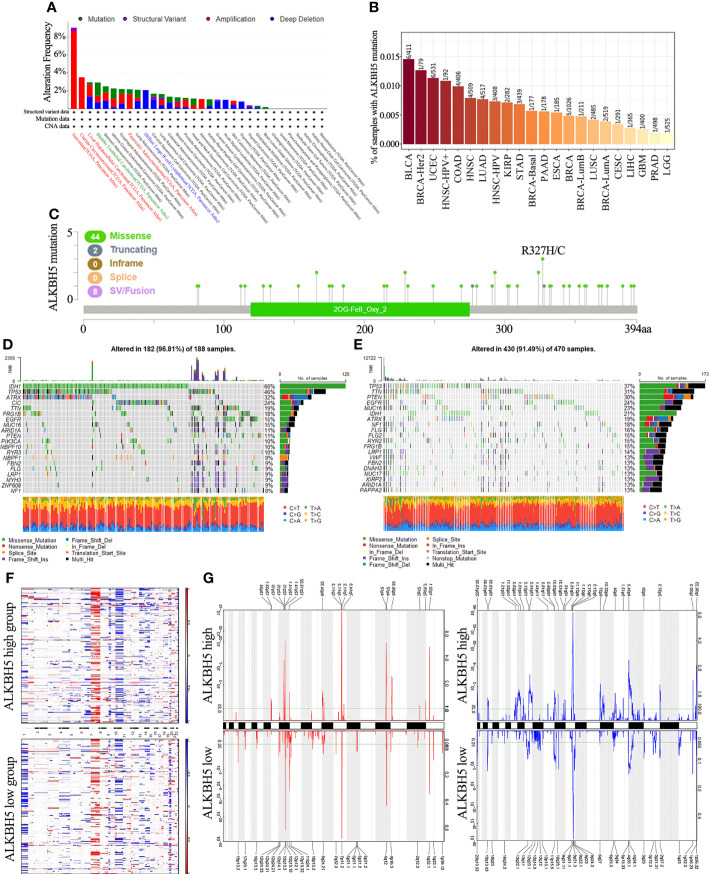
Distinct genomic profiles associated with ALKBH5 expression and integrative analysis of complex cancer genomics and clinical profiles. **(A)** Genetic alteration features (Mutation, Structural Variant, Amplification and Deep Deletion) of ALKBH5 in 32 different tumors were analyzed in TCGA database by the cBioPortal tool. The red label represented the tumors with the top 3 levels of amplification. The green label represented the tumor with the highest mutation level and the blue label represented the tumor with the highest deep deletion level; **(B)** Mutation rates of ALKBH5 gene in various tumors via TIMER portal; **(C)** The mutation sites of ALKBH5 in multiple tumors by the cBioPortal tool. Detection of differential somatic mutations in gliomas, including 25% ALKBH5low group **(D)** and 25% ALKBH5high group **(E)**. Only the top 20 genes with the highest mutation rates were shown; **(F)** The CNAs profile analysis about 25% ALKBH5low group and 25% ALKBH5high group in TCGA dataset via GISTIC2.0; **(G)** Frequency of amplifications and deletions in gliomas with low and high ALKBH5 expression (Blue, deletion; red, amplification).

The authors apologize for this error and state that this does not change the scientific conclusions of the article in any way. The original article has been updated.

## Publisher’s Note

All claims expressed in this article are solely those of the authors and do not necessarily represent those of their affiliated organizations, or those of the publisher, the editors and the reviewers. Any product that may be evaluated in this article, or claim that may be made by its manufacturer, is not guaranteed or endorsed by the publisher.

