# Supplementary figures and images for: Pan-Cancer Analysis Shows That ALKBH5 Is a Potential Prognostic and Immunotherapeutic Biomarker for Multiple Cancer Types Including Gliomas
Source: Front Immunol. 2022 Apr 4;13:849592. doi: 10.3389/fimmu.2022.849592 (PMC9013910; doi:10.3389/fimmu.2022.849592)

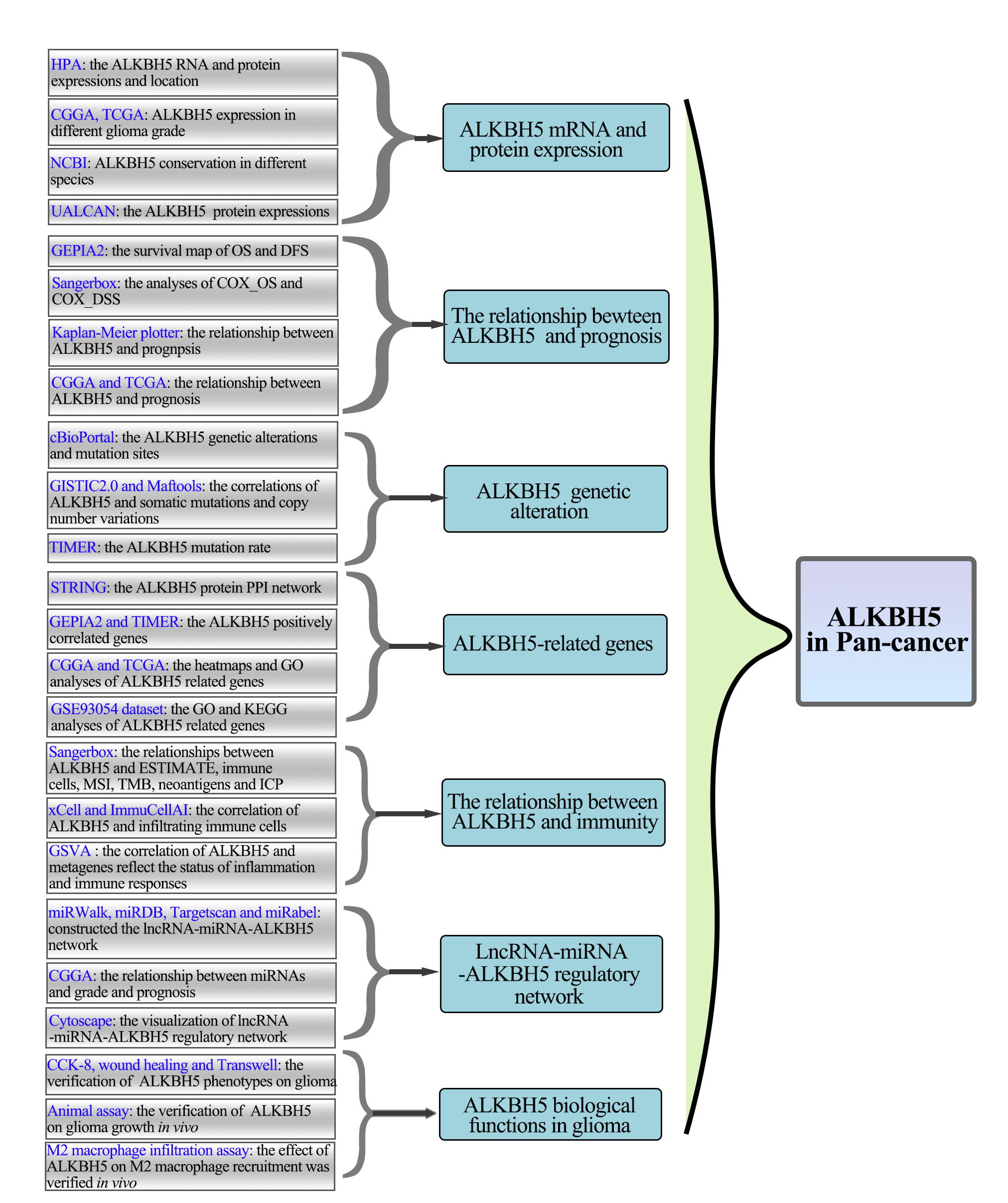

Supplement: Supplementary Figure 1 — Flow chart of bioinformatics analyses. [file DataSheet_1.zip › Supplementary materials/Supplementary Figure 1.tif]

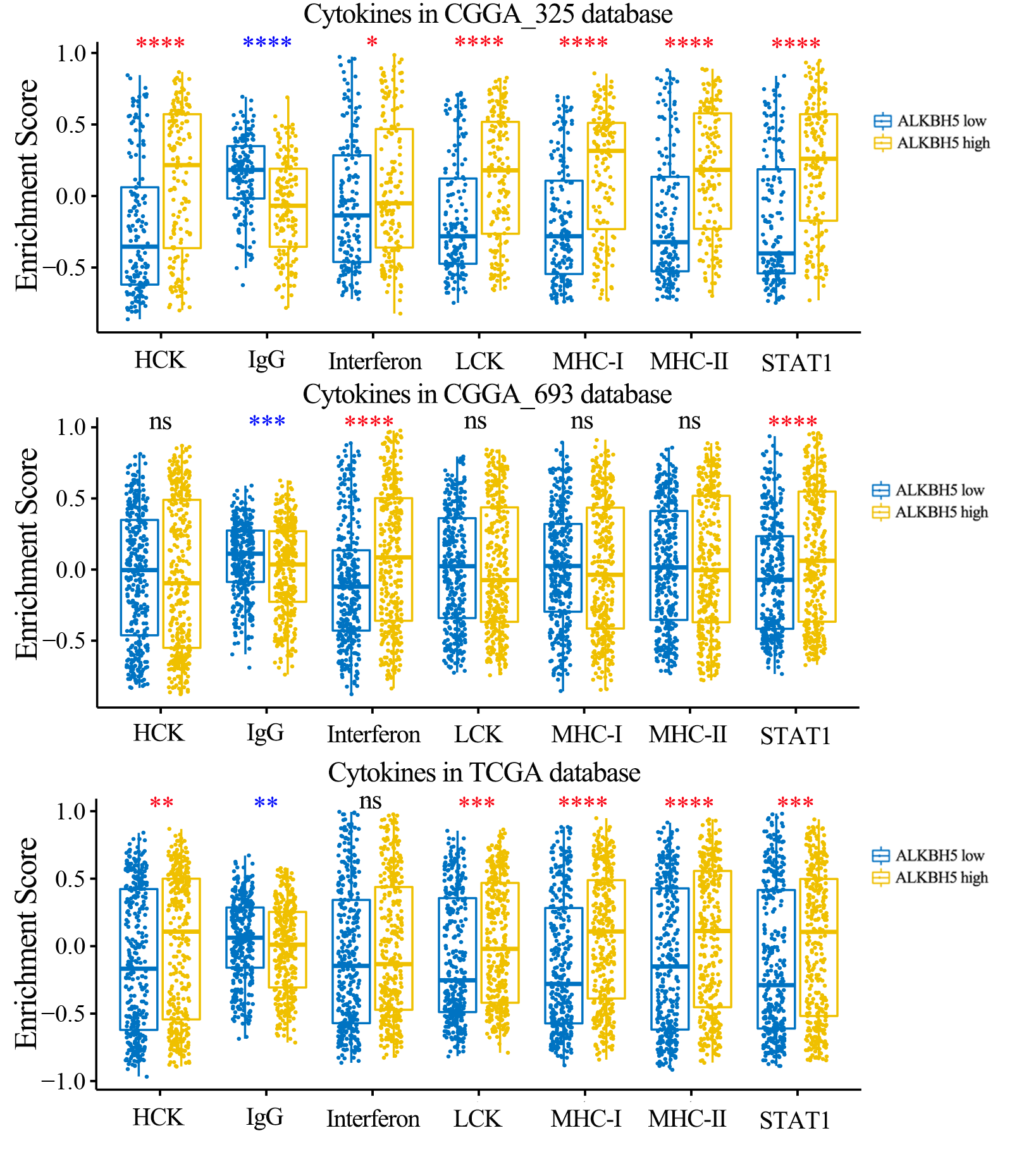

Supplement: Supplementary Figure 1 — Flow chart of bioinformatics analyses. [file DataSheet_1.zip › Supplementary materials/Supplementary Figure 10.tif]

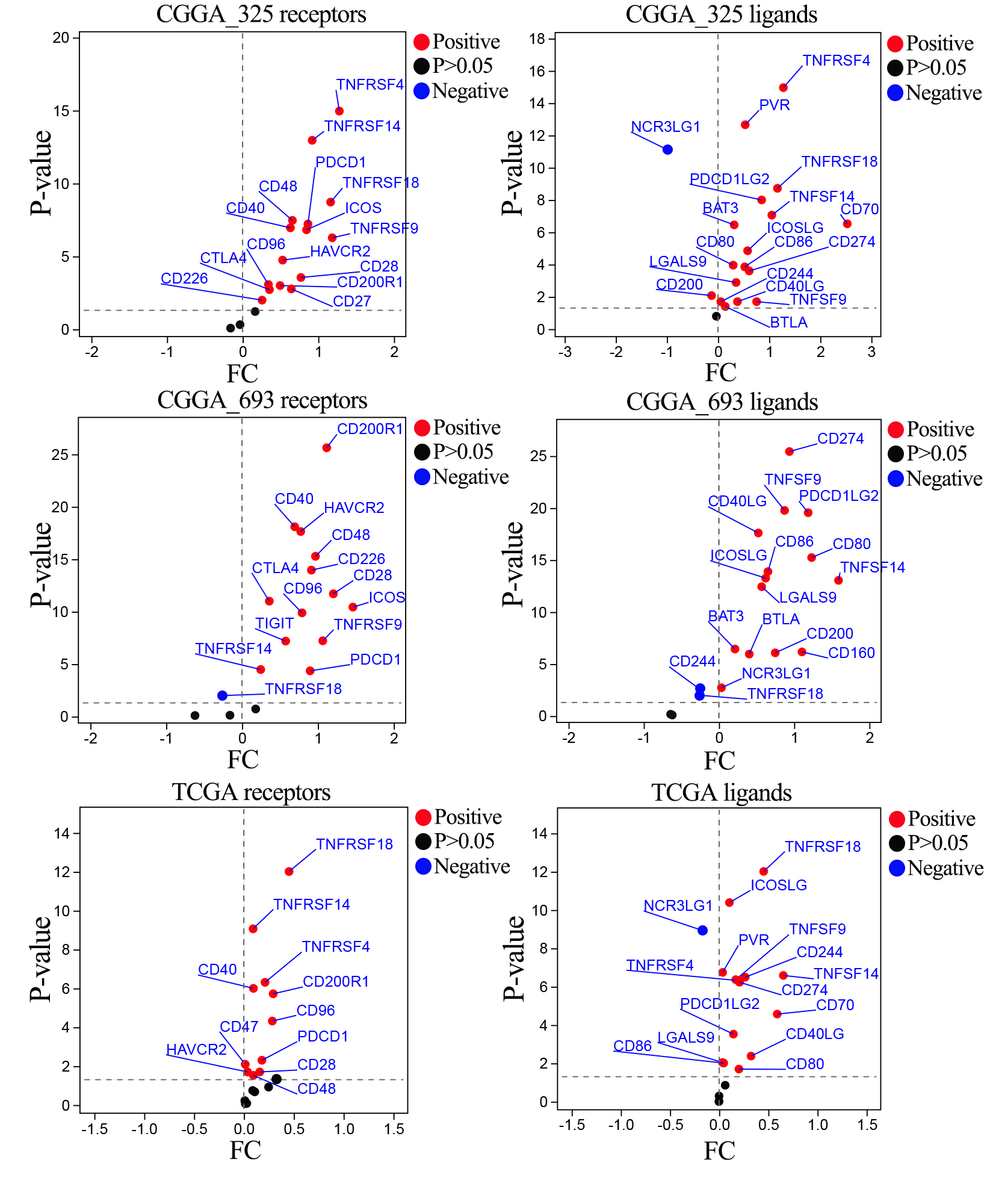

Supplement: Supplementary Figure 1 — Flow chart of bioinformatics analyses. [file DataSheet_1.zip › Supplementary materials/Supplementary Figure 11.tif]

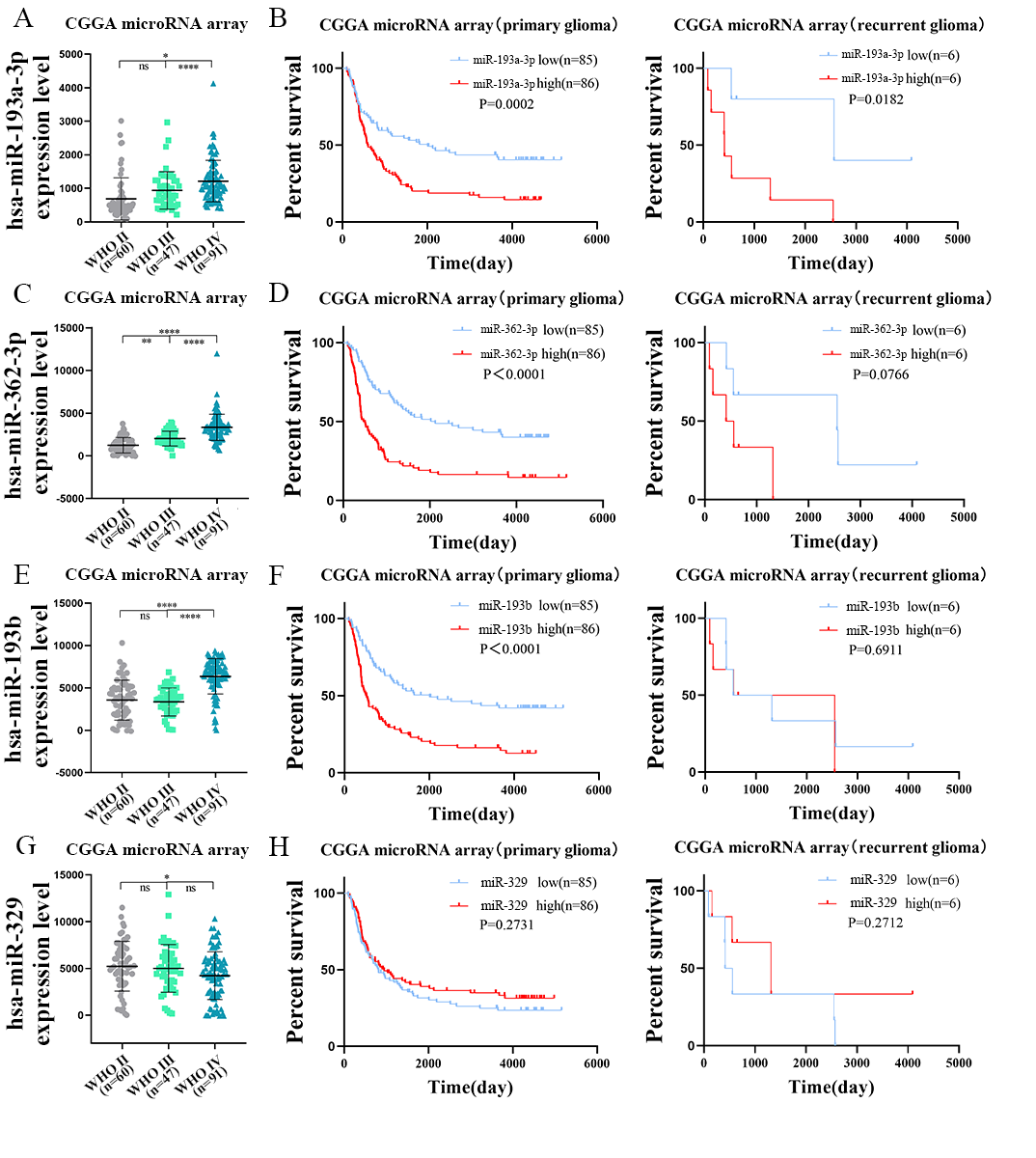

Supplement: Supplementary Figure 1 — Flow chart of bioinformatics analyses. [file DataSheet_1.zip › Supplementary materials/Supplementary Figure 12.tif]

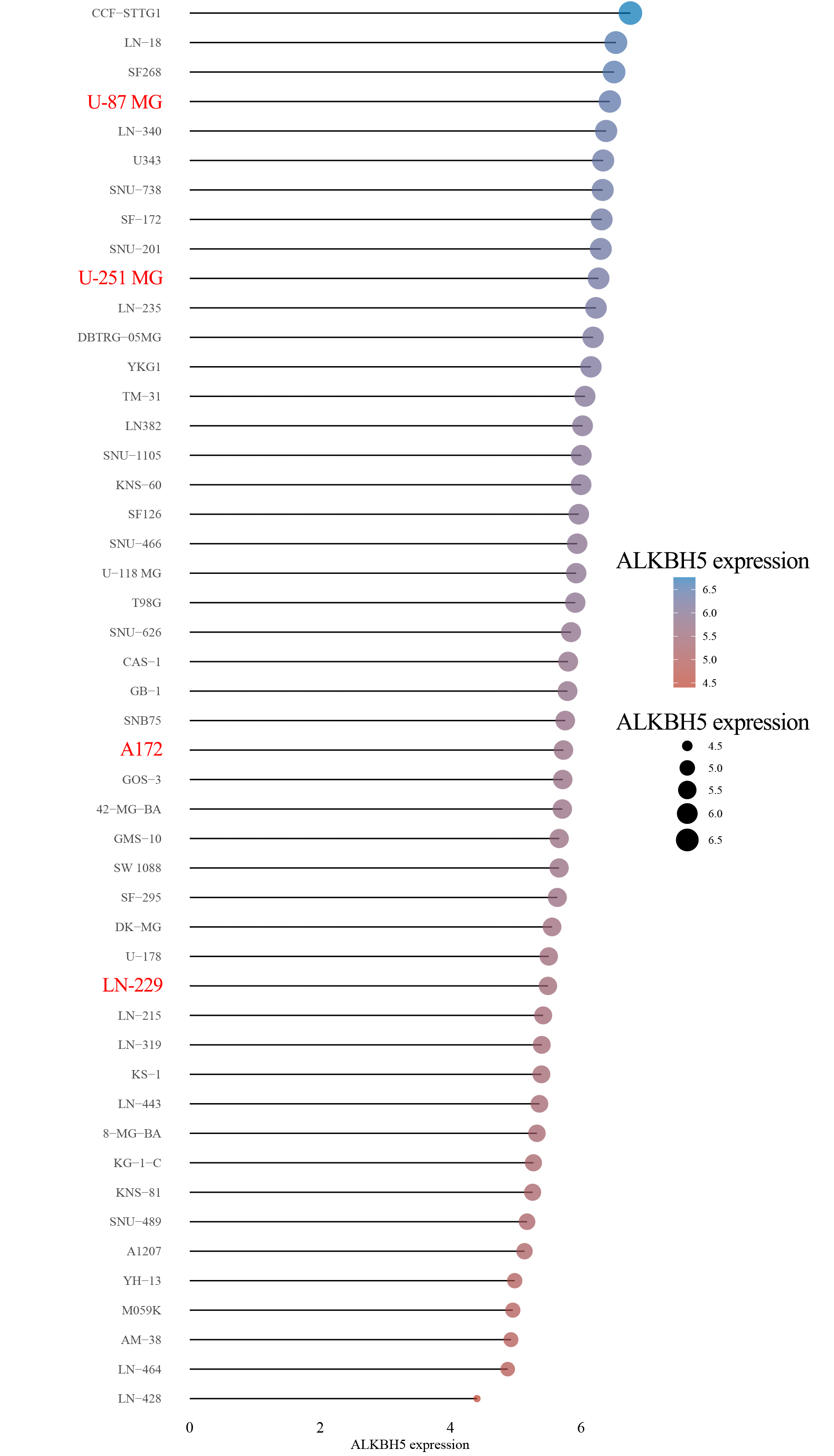

Supplement: Supplementary Figure 1 — Flow chart of bioinformatics analyses. [file DataSheet_1.zip › Supplementary materials/Supplementary Figure 13.tif]

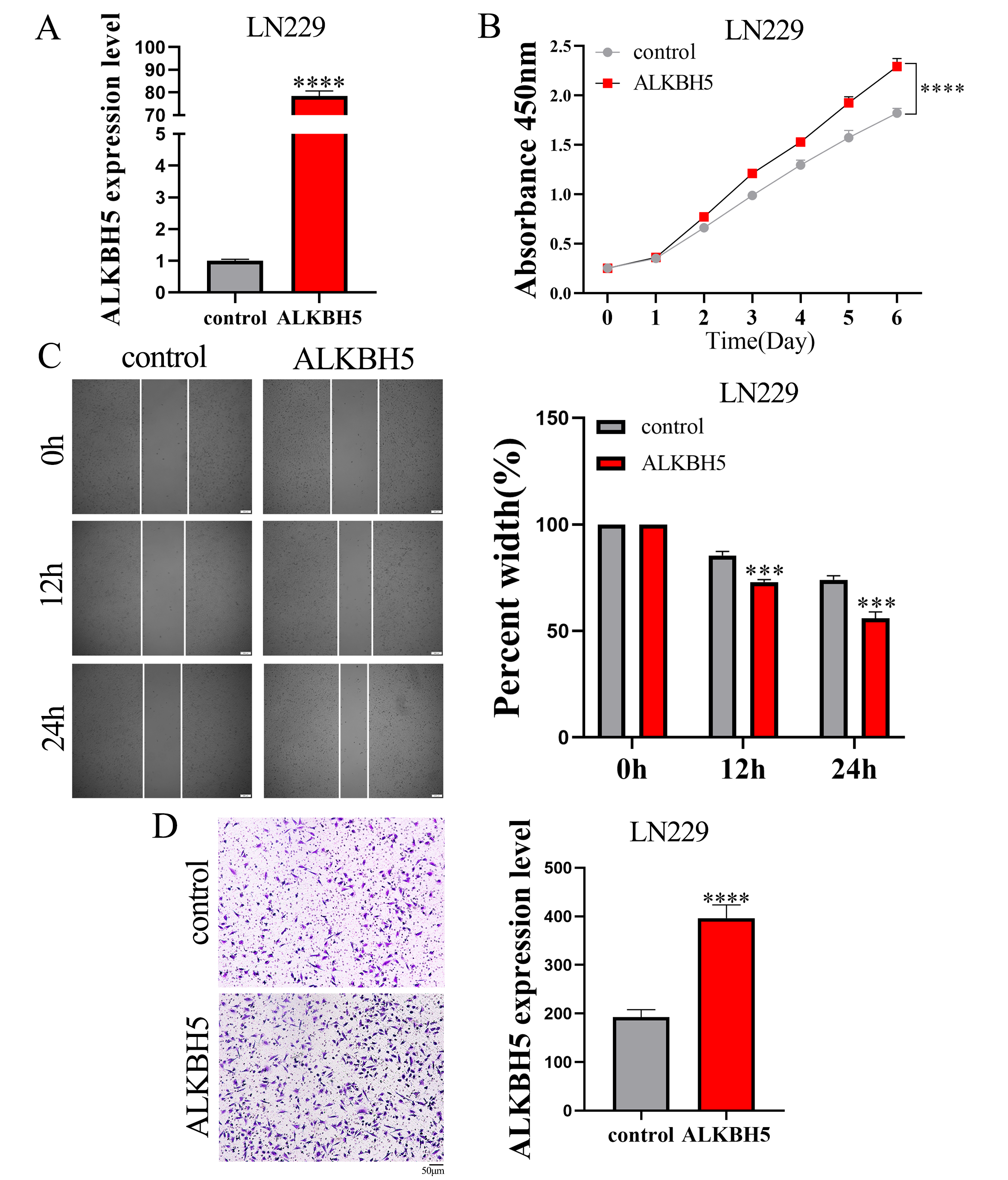

Supplement: Supplementary Figure 1 — Flow chart of bioinformatics analyses. [file DataSheet_1.zip › Supplementary materials/Supplementary Figure 14.tif]

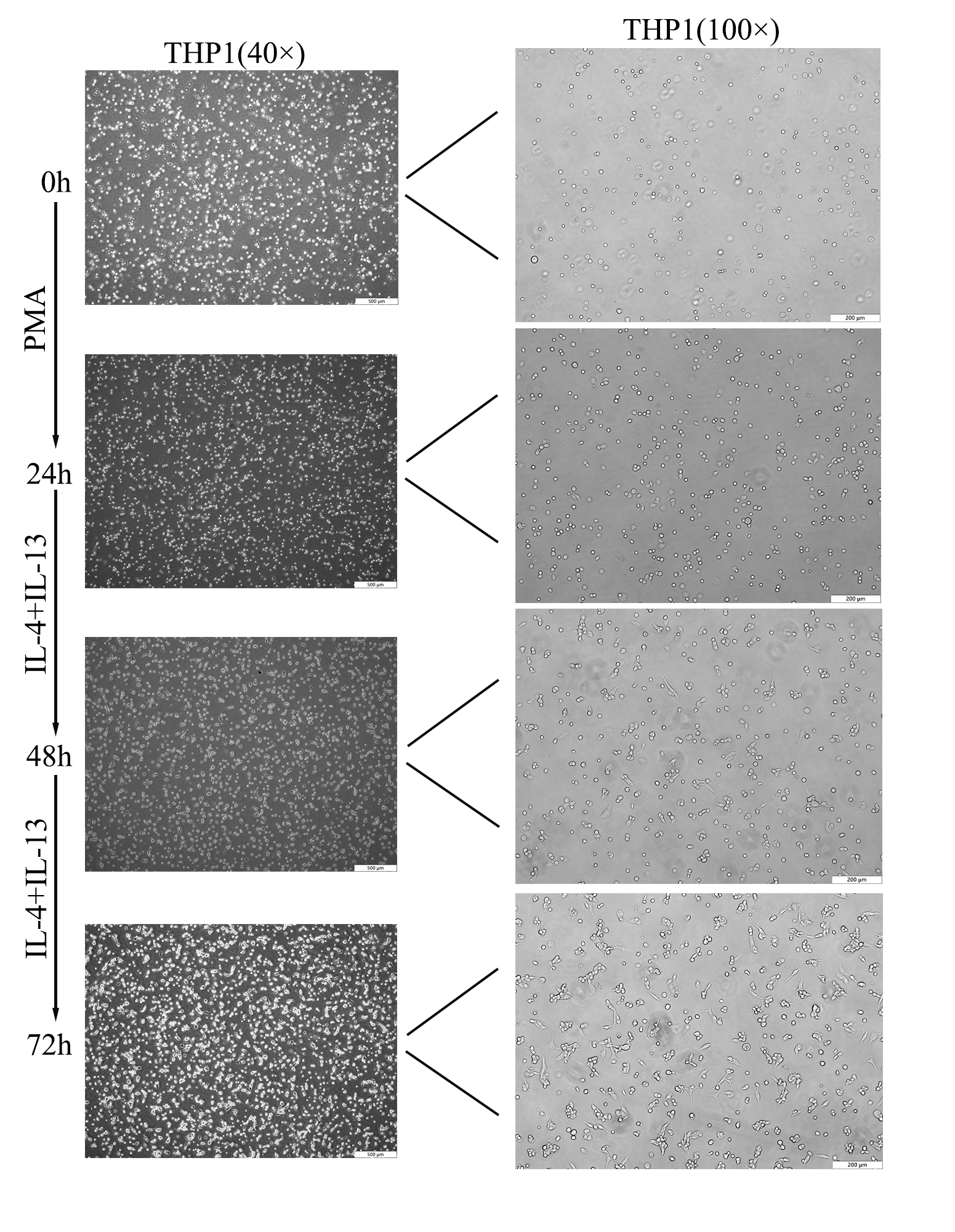

Supplement: Supplementary Figure 1 — Flow chart of bioinformatics analyses. [file DataSheet_1.zip › Supplementary materials/Supplementary Figure 15.tif]

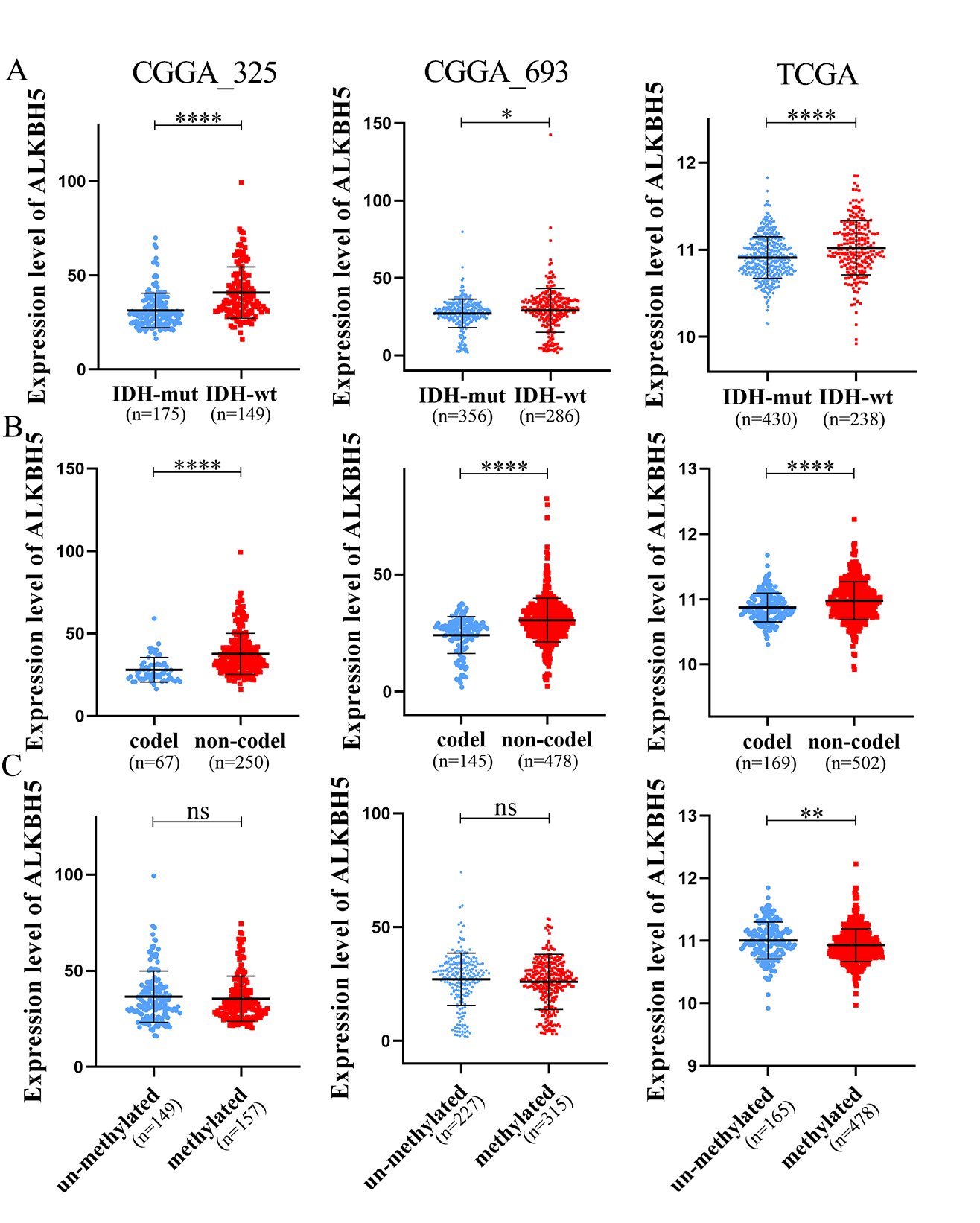

Supplement: Supplementary Figure 1 — Flow chart of bioinformatics analyses. [file DataSheet_1.zip › Supplementary materials/Supplementary Figure 2.tif]

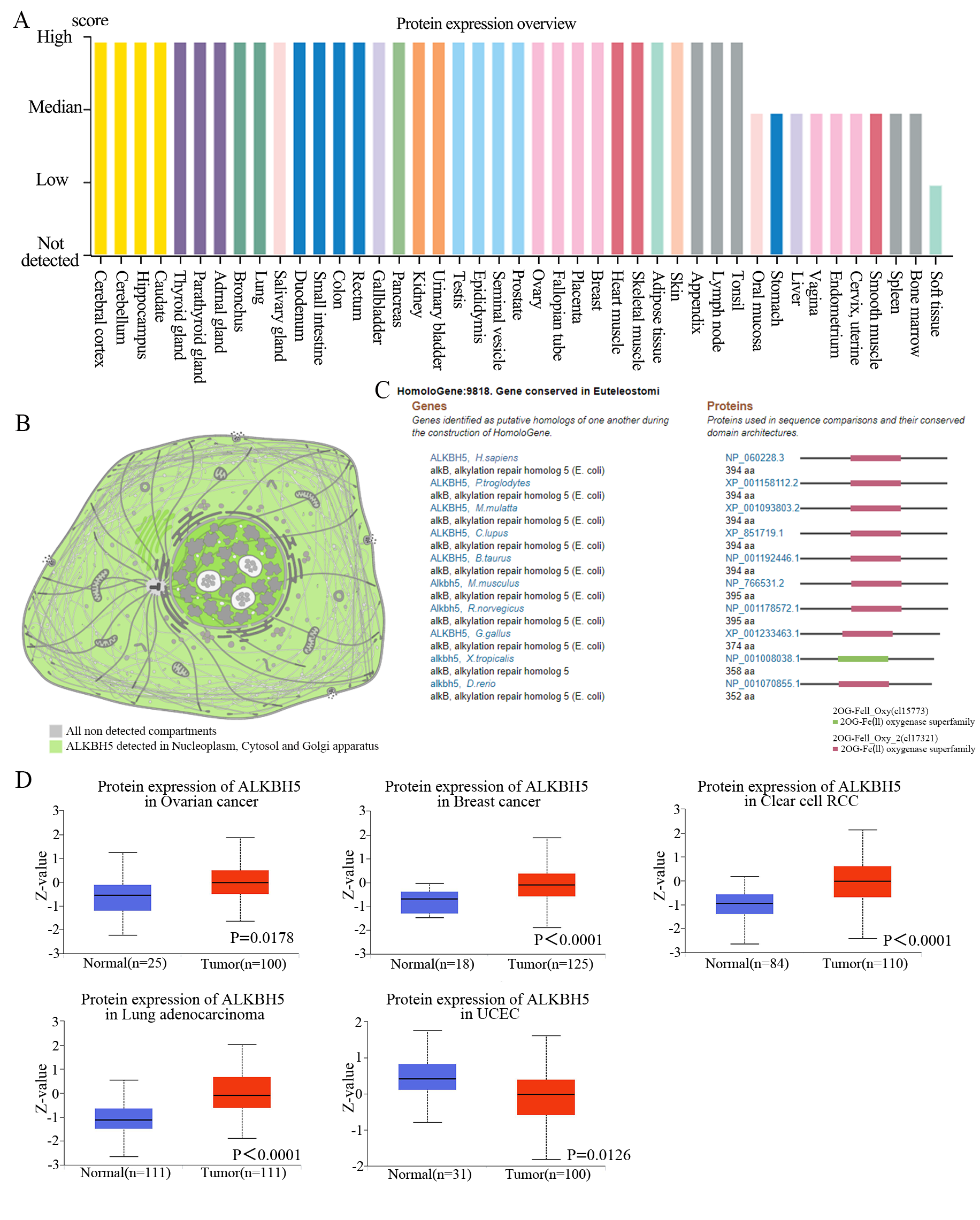

Supplement: Supplementary Figure 1 — Flow chart of bioinformatics analyses. [file DataSheet_1.zip › Supplementary materials/Supplementary Figure 3.tif]

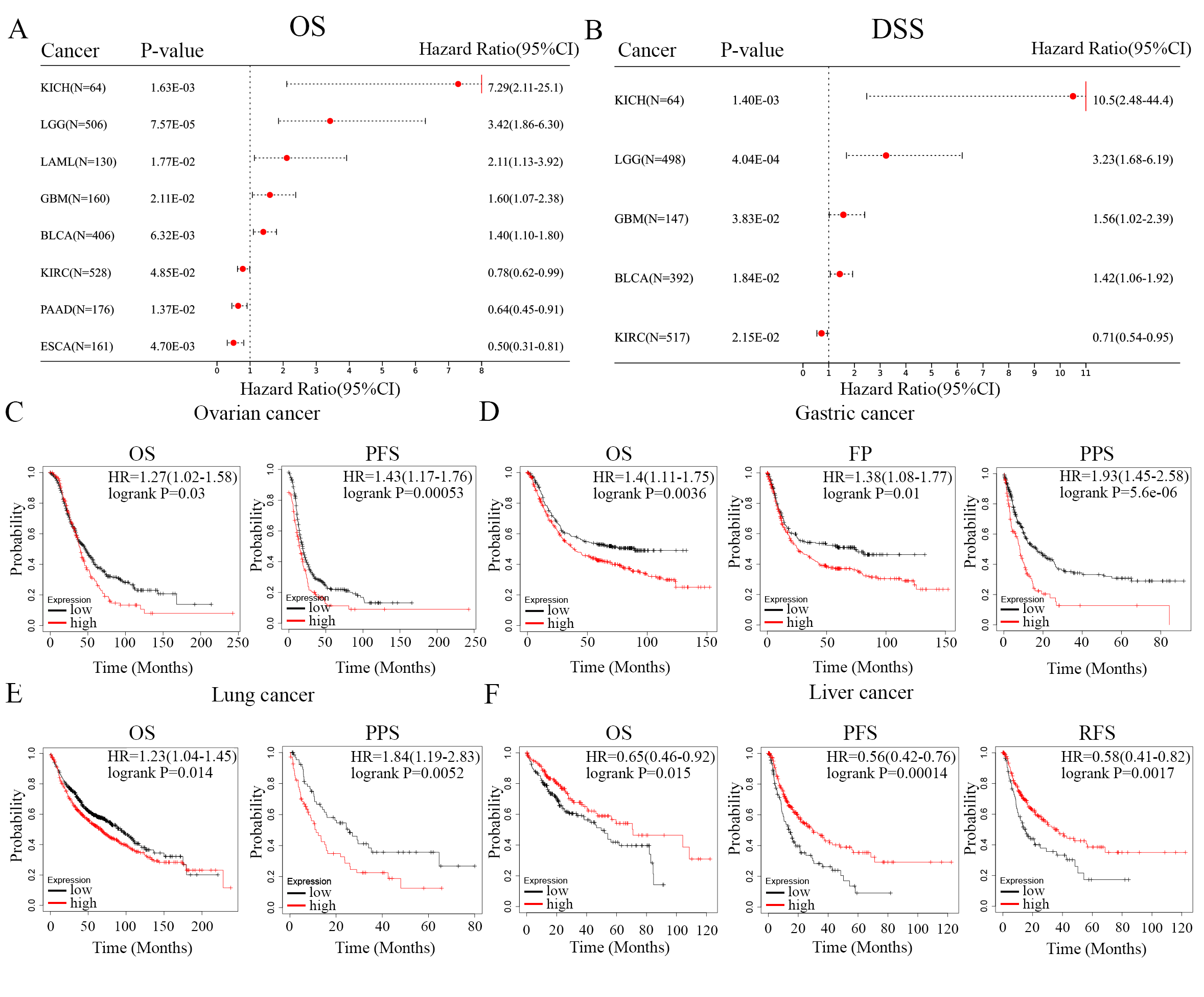

Supplement: Supplementary Figure 1 — Flow chart of bioinformatics analyses. [file DataSheet_1.zip › Supplementary materials/Supplementary Figure 4.tif]

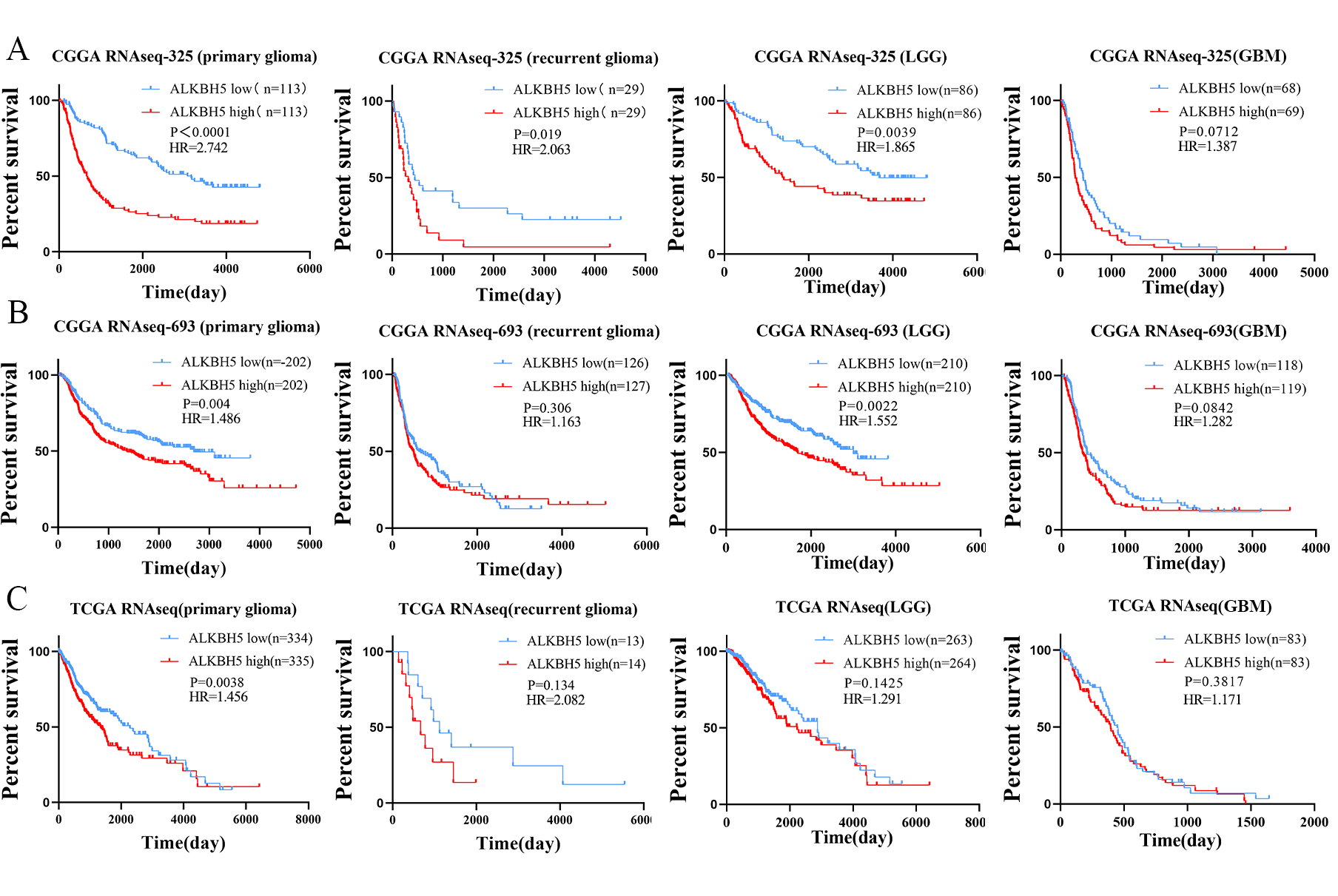

Supplement: Supplementary Figure 1 — Flow chart of bioinformatics analyses. [file DataSheet_1.zip › Supplementary materials/Supplementary Figure 5.tif]

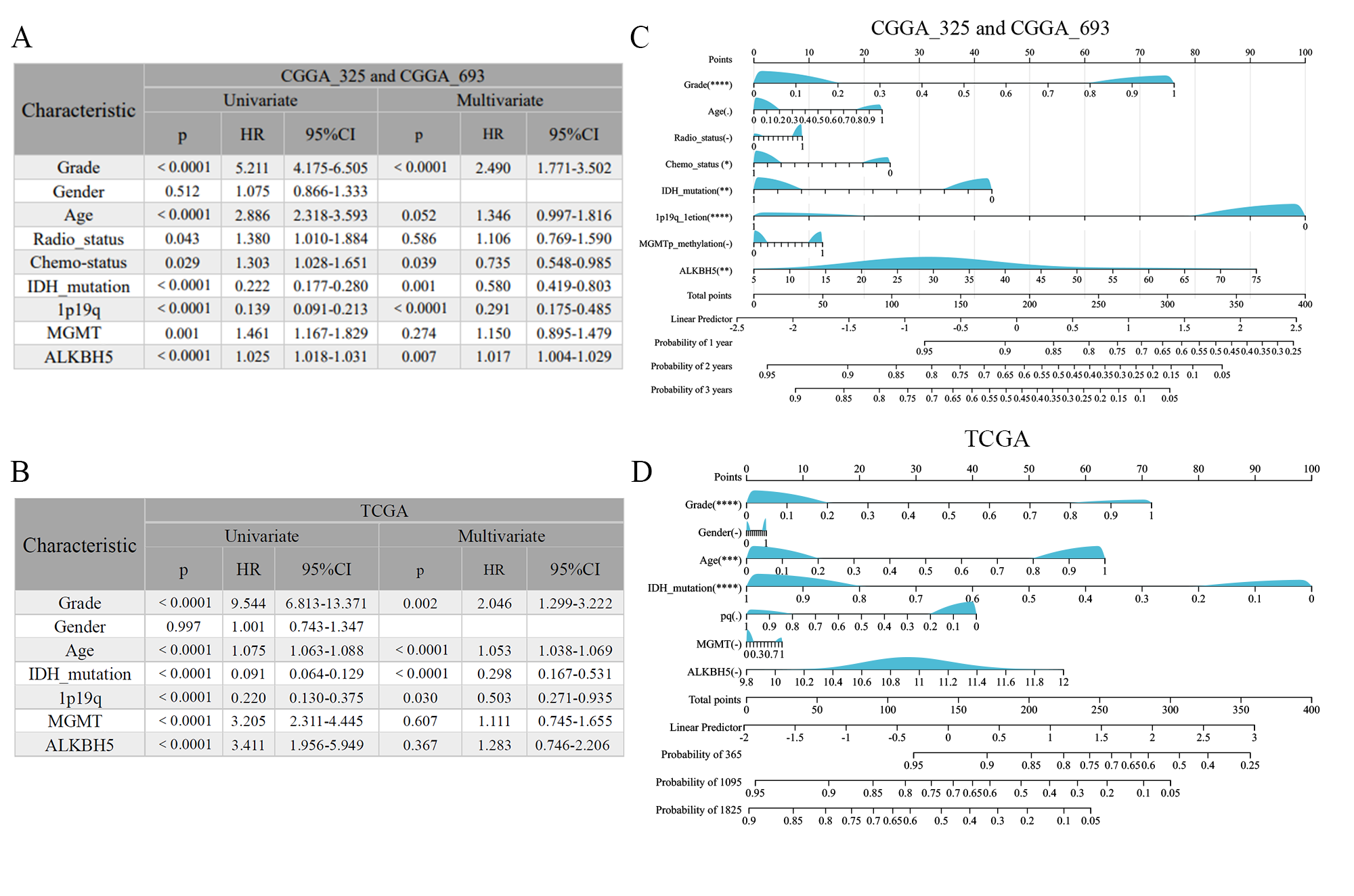

Supplement: Supplementary Figure 1 — Flow chart of bioinformatics analyses. [file DataSheet_1.zip › Supplementary materials/Supplementary Figure 6.tif]

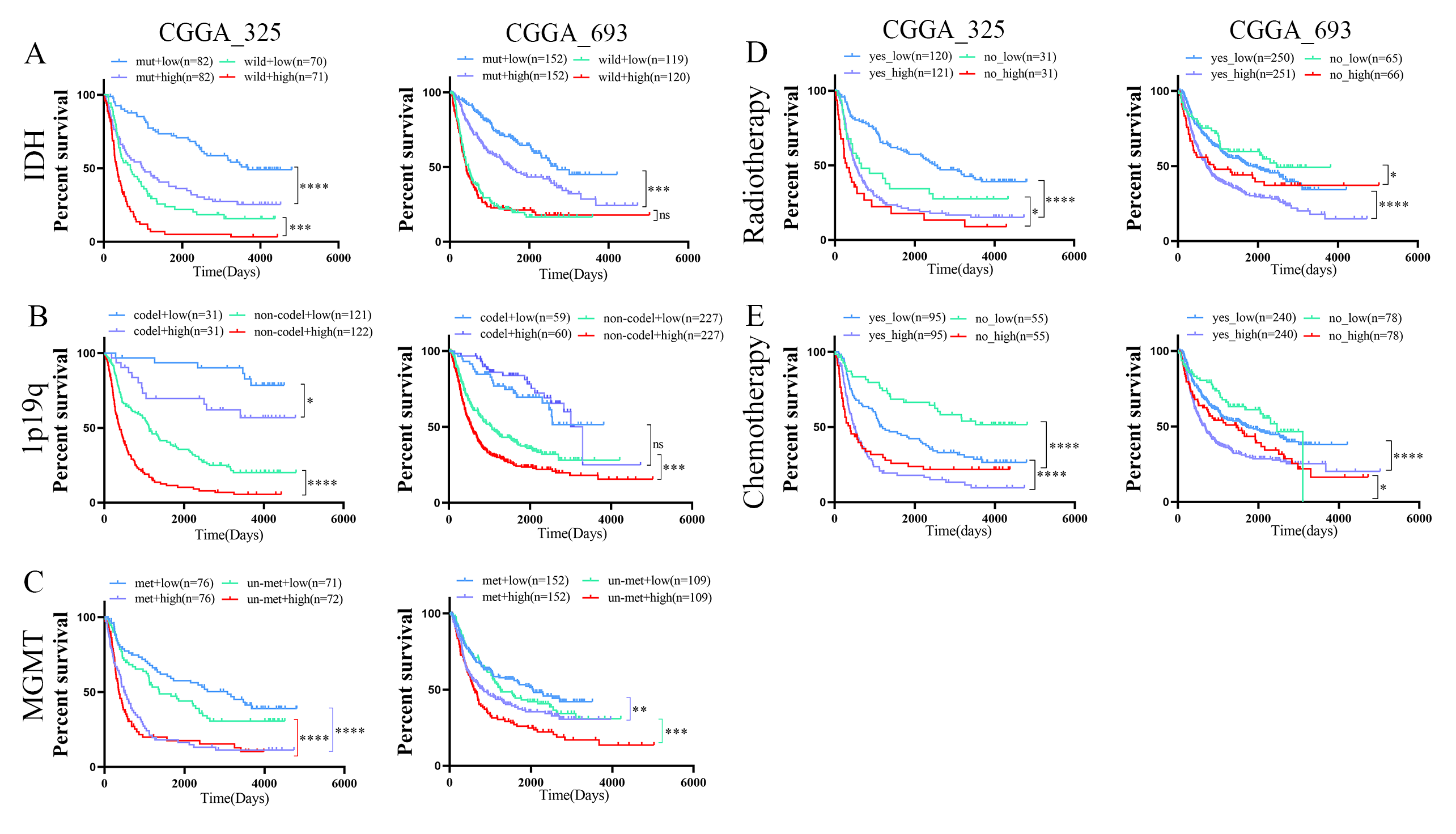

Supplement: Supplementary Figure 1 — Flow chart of bioinformatics analyses. [file DataSheet_1.zip › Supplementary materials/Supplementary Figure 7.tif]

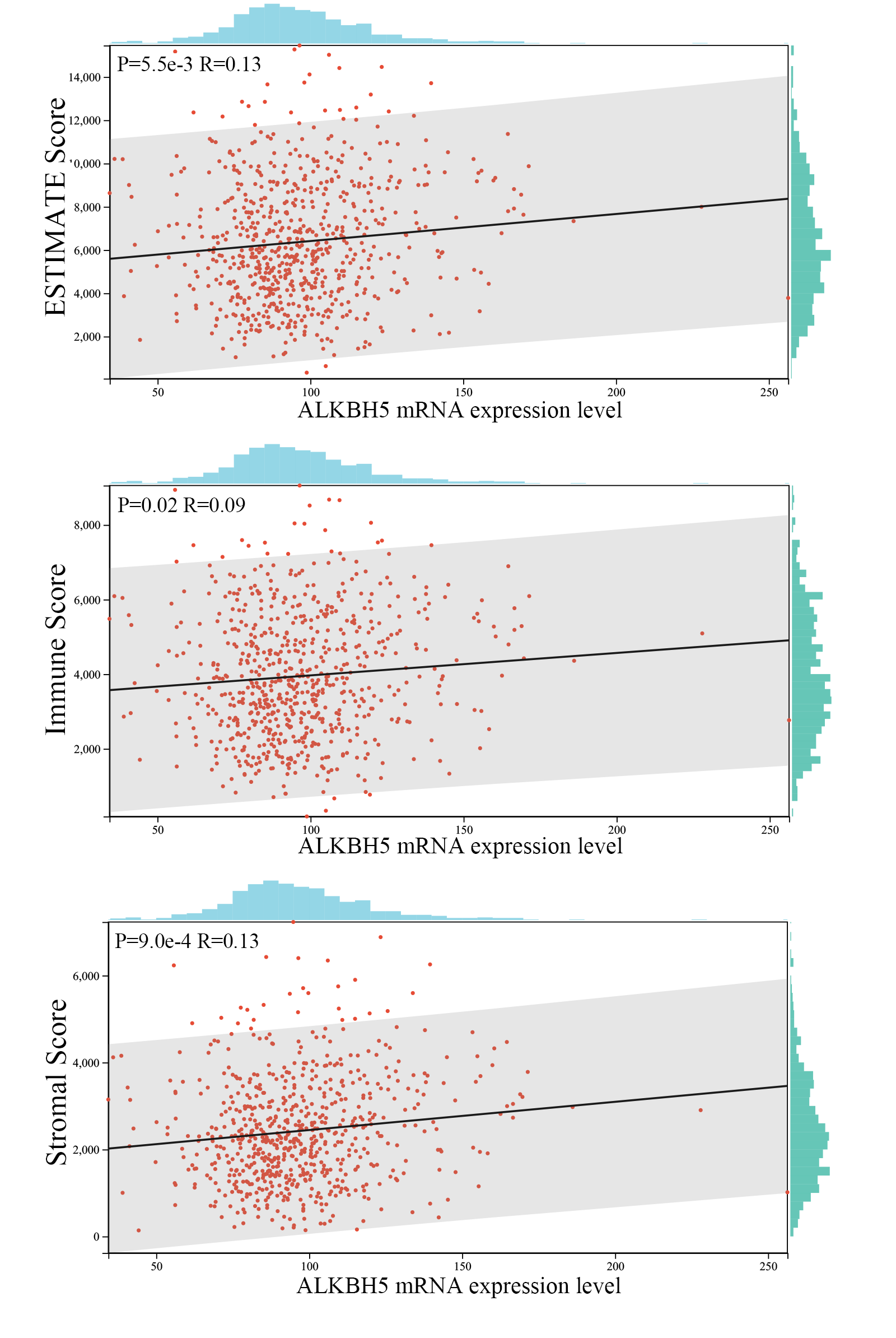

Supplement: Supplementary Figure 1 — Flow chart of bioinformatics analyses. [file DataSheet_1.zip › Supplementary materials/Supplementary Figure 8.tif]

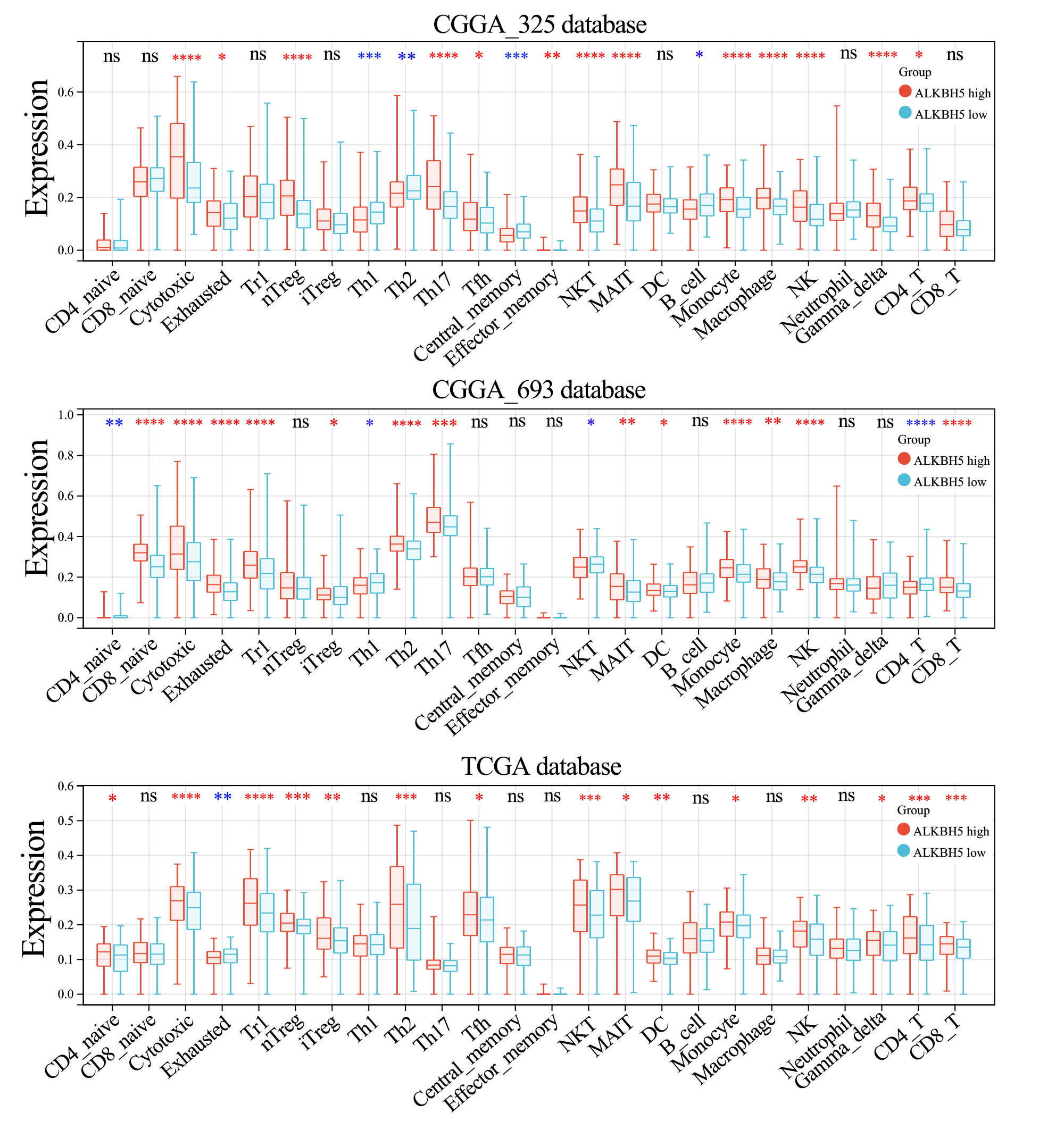

Supplement: Supplementary Figure 1 — Flow chart of bioinformatics analyses. [file DataSheet_1.zip › Supplementary materials/Supplementary Figure 9.tif]
